# Multitarget Generate Electrolyte Additive for Lithium Metal Batteries

**DOI:** 10.1002/adma.202502086

**Published:** 2025-06-18

**Authors:** Xiangyang Liu, Jianchun Chu, Sa Xue, Daquan Wang, Zhuoyang Lu, Meng Zhang, Yongqi Liu, Xin Xu, Yilin Zhang, Jiangang Long, Lingjie Meng, Jiayin Yuan, Maogang He

**Affiliations:** ^1^ Key Laboratory of Thermal Fluid Science and Engineering of MOE School of Energy and Power Engineering Xi'an Jiaotong University Xi'an 710049 China; ^2^ School of Chemistry Xi'an Jiaotong University Xi'an 710049 China; ^3^ Key Laboratory of Biomedical Information Engineering of MOE School of Life Science and Technology Xi'an Jiaotong University Xi'an 710049 China; ^4^ Department of Chemistry Stockholm University Svante Arrheniusväg 16C Stockholm 106 91 Sweden; ^5^ Applied Science and Technology Graduate Group University of California Berkeley CA 94720 USA; ^6^ California Institute for Quantitative Biosciences University of California Berkeley CA 94720 USA

**Keywords:** artificial intelligence, electrolyte, generative model, lithium battery, molecular design

## Abstract

Electrolyte additives are crucial for accelerating the commercialization of lithium metal batteries (LMBs), yet designing effective additives is challenging due to the need to balance conflicting properties, such as eectrochemical performance and nonflammability. To address this challenge, a deep learning‐assisted generative model is developed for multiobjective optimization of electrolyte additives. Overcoming data scarcity, the dataset is expanded using a molecular categorization derivation method, increasing single‐property data points to 70 095 multiproperty data points. Coupled with an asynchronous limited decoder and adversarial regulation strategy for latent distribution, this approach achieved 100% generative efficiency for structurally complex and diverse molecules in vast chemical space. The method is validated by discovering 2,4‐bis(2‐fluoroethoxy) tetrafluorocyclotriphosphazene (DFEPN), a novel additive with excellent flame resistance and stable dual electrode/electrolyte interphases. In a Li||LiFePO_4_ full cell with a commercial electrolyte, DFEPN enables an order of magnitude increase in capacity retention, outperforming the state‐of‐the‐art flame‐retardant additive ethoxy(pentafluoro)cyclotriphosphazene by 33%. This study offers a pathway for developing safe and reliable lithium battery electrolytes, particularly under severe data constraints, and has broader implications for advanced battery design.

## Graphical abstract

This study presents a deep learning‐assisted generative model for electrolyte additives in lithium metal batteries (LMBs). The approach overcomes data scarcity by proposing the molecular categorization method and achieves 100% generative efficiency. The model led to the discovery of 2,4‐bis(2‐fluoroethoxy) tetrafluorocyclotriphosphazene, a flame‐resistant additive that significantly improves capacity retention, offering a promising solution for safe and high‐performance LMBs.

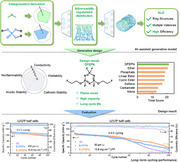

## 1 Introduction

Lithium metal batteries (LMBs) have struggled for widespread adoption due to inherent challenges, including flammability and explosive tendencies.^[^ ^1^, ^2^, ^3^ ^]^ Decades after being invented, LMBs have finally culminated in the replacement by comparably safer lithium‐ion batteries.^[^ ^4^ ^]^ However, the immense potential of LMBs for unparalleled energy density has continually captivated researchers, establishing them as the “Holy Grail” of next‐generation secondary battery chemistries.^[^ ^5^ ^]^

During the century‐long attempt to commercialize LMBs, exploring and applying non‐flammable additives is the most convenient and efficient strategy for addressing safety issues.^[^ ^2b^ ^]^ However, the state‐of‐the‐art electrolyte additives failed to meet diverse requirements in both non‐flammability and high electrochemical performance, including reduction stability, adequate electrode wettability, and efficient ion transport.^[^ ^6^ ^]^ Conventional design of electrolyte additives through trial‐and‐error is hindered by limited understanding of the complex interplay between molecular structure and multiple, often conflicting, properties. For example, common fire‐retardant additives such as trimethyl phosphate and triphenyl phosphate enhance fire resistance at the cost of battery capacity and lifespan. While ethoxy(pentafluoro)cyclotriphosphazene (PFPN) serves as a representative multifunctional additive by acting as both a flame retardant and a cathode/electrolyte interphase stabilizer, it has limited contribution to overcome the unstable anode/electrolyte interphase.^[^ ^7^ ^]^

Generative deep learning has recently established a different path to molecular design by combining molecular representation, generation, and optimization,^[^ ^8^ ^]^ thereby overcoming limitations of current chemical databases through the generation of novel data samples that resemble training data distributions.^[^ ^9^ ^]^ This capability enables the atomic‐scale design of novel molecular structures with optimal properties across vast chemical spaces, accelerating material discovery more efficiently and effectively.^[^ ^10^ ^]^ Despite such advance, no generative deep learning models currently address multiproperty optimization for electrolytes; present approaches are restricted to predicting single properties, e.g., Coulombic efficiency (CE) or reduction stability.^[^ ^11^ ^]^ Two major challenges in our view hinder progress: 1) Unlike drug discovery,^[^ ^12^ ^]^ where extensive datasets are available, (e.g., ChEMBL^[^ ^13^ ^]^ with 2.4 million and DrugBank^[^ ^14^ ^]^ with 500 000 compounds), the structure‐performance datasets for lithium battery electrolytes are very confined, complicating critical feature extraction and increasing the risk of overfitting. 2) The trade‐offs between multiple electrolyte properties make the optimization function discontinuous, reducing generative accuracy and efficiency.

**Figure 1:**
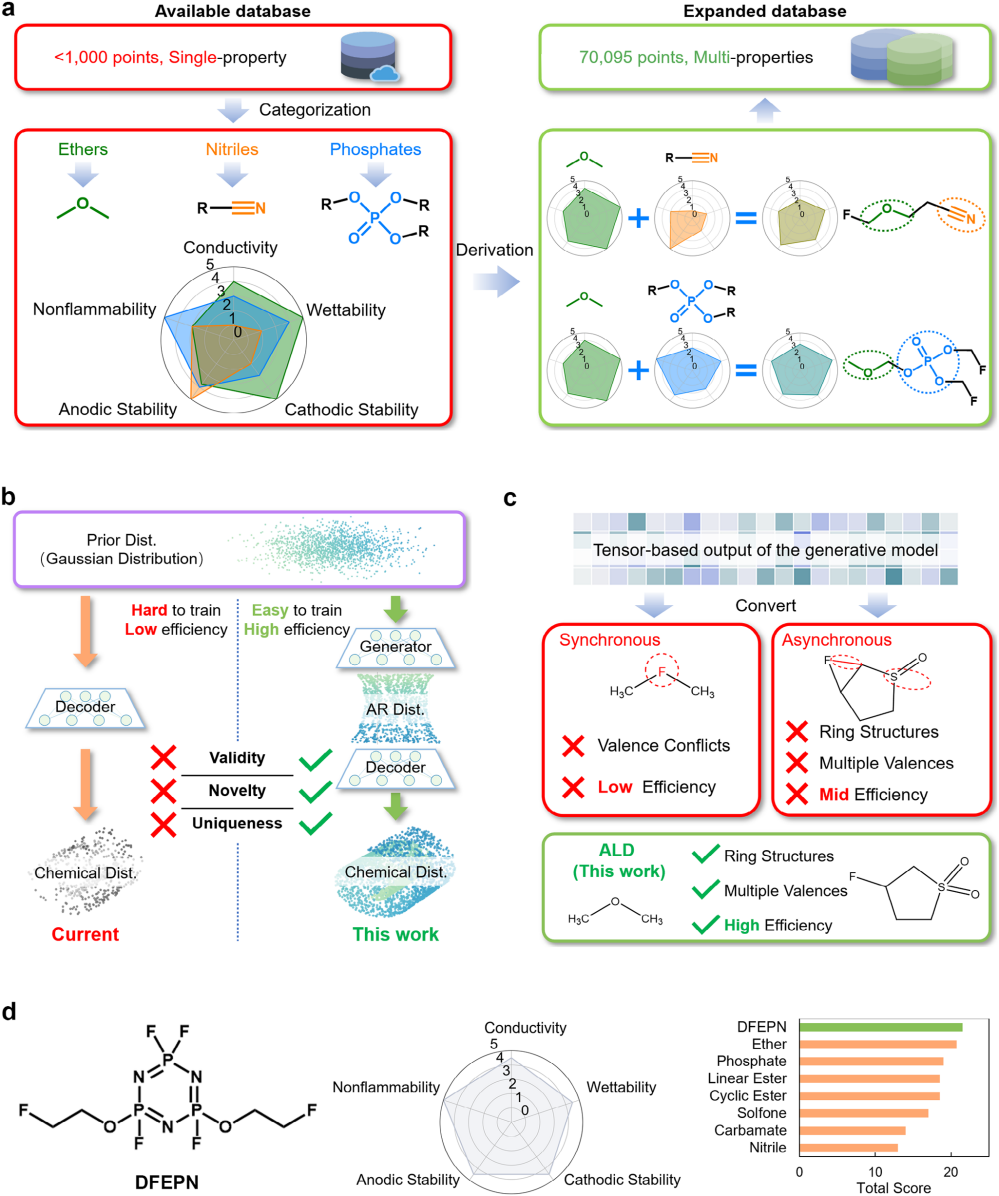
Generative model illustration and designed result. a) Workflow for creating a structure‐performance database. b) Comparison between current and our distributional transformation process. c) Comparison of ALD and asynchronous/asynchronous. d) Molecular structure and performance of DFEPN.

In this study, we develop a generative deep learning model to achieve high‐efficiency, simultaneous multiproperty design of electrolyte additives. To address the challenge of data deprivation, we construct a robust dataset by proposing and applying a molecular categorization derivation method (**Figure** **1**a, further details provided in the Supporting Information). This method allows to systematically evaluate structure‐performance relationships for 14019 molecules selected from 741144 molecules in the ZINC database (DB),^[^ ^15^ ^]^ based on criteria of fewer than 30 heavy atoms, at least one atom of F, P, or S, and at least one characteristic functional group to balance practicality, relevance, and computational efficiency. The generative model's performance depends on efficient conversion between a sampling‐friendly distribution and the complex molecular structure distribution.^[^ ^16^ ^]^ Here, we replace the conventional manually set prior distribution (often Gaussian) with a trainable, adversarially regularized distribution (Figure 1b), achieving more effective chemical space embedding. This approach simplifies the distributional transformation process during training and enhances sampling performance (100% validity, 68.8% uniqueness, and 100% novelty; see the Supporting information for details of the evaluation framework definition^[^ ^17^ ^]^). To convert the tensor‐based output of the generative model into molecular structures in a graph form, two decoding methods are typically employed: synchronous decoding^[^ ^18^ ^]^ and asynchronous decoding.^[^ ^19^ ^]^ Synchronous decoding samples all nodes (atoms) and edges (bonds) simultaneously, as in the basic graph variational autoencoder (GVAE) model,^[^ ^18^ ^]^ but leads to valence conflicts and generative efficiency reduction (e.g., in Figure 1c an O atom is erroneously converted into F). Asynchronous decoding samples one node at a time, with valence constraints.^[^ ^20^ ^]^ By asynchronous decoding, some valence issues are addressed but specific cases remain unresolved: such as ring structures or atoms with multiple common valences, which can sample additional erroneous bonds (e.g., assigning extra bonds to S and adding an extra bond to the ring). Building on asynchronous decoding, the GVAE‐Monte Carlo tree search (MCTS) model partially resolves ring atom conflicts, but issues with multivalence atoms remain.^[^ ^21^ ^]^ Moreover, junction tree‐VAE (JTVAE), a synchronous decoding variant, improves validity by representing groups instead of individual atoms as graph nodes, at the cost of diversity in generated molecules.^[^ ^22^ ^]^ To balance generative efficiency with the search scope, we introduce an enhanced asynchronous limited decoder (ALD), which offers two main advantages to avoid the asynchronous decoding problem: 1) prior to sampling bonds for an atom, the maximum‐likelihood valence is calculated, and 2) edges are sampled on the basis of both start and end nodes concurrently. The resulting generative model, termed the Adversarially Regularized E(n)‐Equivariant Conditional Variational Graph Autoencoder (ARECVGA), integrates an attention E(n)‐Equivariant Graph Neuron Network (AEGNN) for robust molecular structure representation, significantly outperforms the available models such as GraphVAE,^[^ ^18^ ^]^ MolGAN,^[^ ^23^ ^]^ and NAGVAE^[^ ^24^ ^]^ in validity, uniqueness and novelty (see the Supporting Information for details).

The optimized output molecule, 2,4‐bis(2‐fluoroethoxy) tetrafluorocyclotriphosphazene (DFEPN), demonstrates the highest overall performance under demanded conditions (Figure 1d). DFEPN shows marked improvements both in non‐flammability and electrochemical performance as an additive to the commercial electrolyte (1.0 m LiPF_6_ in ethylene carbonate (EC)/dimethyl carbonate (DMC) (1:1, by vol), abbr. G2) for LMBs.

## 2 Results and Discussion

The structural configuration of DFEPN, featuring a cyclophosphazene backbone enriched with phosphorus atoms analogous to PFPN, inherently demonstrates superior flame‐retardant capabilities, as extensively documented in advanced electrolyte studies.^[^ ^25^ ^]^ Notably, the F atom of ‐CF in DFEPN introduces enhanced electron‐withdrawing effects compared to PFPN, effectively lowering the solvent molecules' lowest unoccupied molecular orbital (LUMO) energy level.^[^ ^26^ ^]^ This optimized electronic structure facilitates the kinetically preferential reduction of DFEPN at the lithium metal anode interface during initial cycling, enabling the in situ formation of a fluorine‐rich solid electrolyte interphase (SEI). Such interfacial engineering synergistically combines exceptional flame suppression performance with remarkable electrochemical stability.

**Figure 2:**
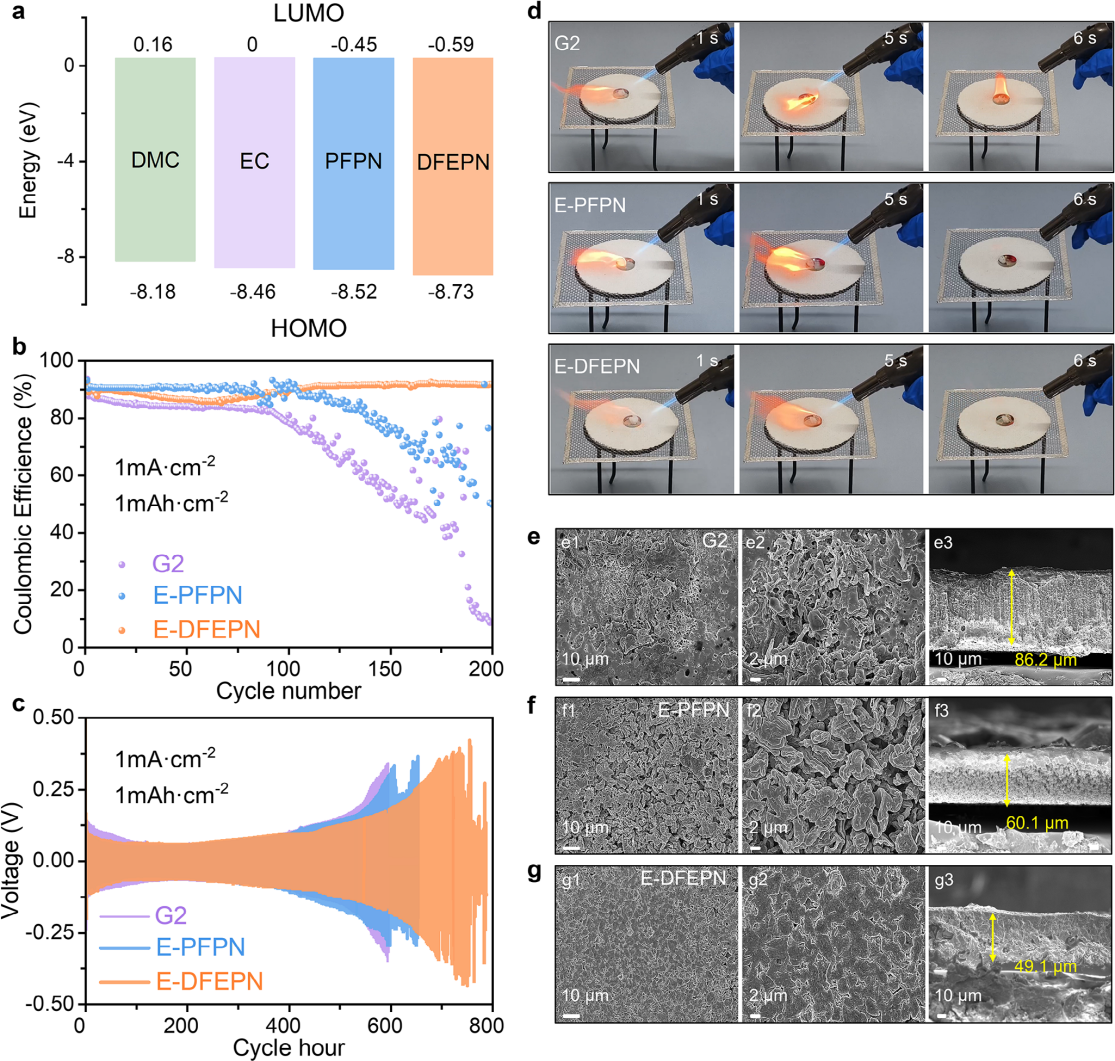
Physicochemical properties and electrochemical performance of LMA. a) LUMOs and HOMOs of four types of electrolyte molecules in this study. b) Long‐term Li deposition/stripping CE of different electrolytes in Li/Cu half cells at 1.0 mA·cm^−2^ and 1.0 mAh·cm^−2^. c) Cycling behavior of Li/Li symmetrical cells with different electrolytes at 1.0 mA·cm^−2^ and 1.0 mAh·cm^−2^. d) Ignition experiments with different electrolytes. e–g) Surface and cross‐sectional morphology of Li deposited on Cu after 100 cycles in G2 (e), E‐PFPN (f) and E‐DFEPN (g).

The LUMO and highest occupied molecular orbital (HOMO) energies of different molecules were calculated by DFT (**Figure** **2**a). The higher F atom content in DFEPN results in a lower LUMO energy than PFPN, indicating that DFEPN is more likely to undergo reduction at the lithium metal anode (LMA) and contribute to the formation of SEI. Previous studies indicate that PFPN as the electrolyte additive at a concentration of 5 wt.% simultaneously delivered safety and electrochemical stability, which was supported by Figures S18 and S19 (Supporting Information).^[^ ^2b^ ^]^ For fair comparison, both PFPN and DFEPN in this study were standardized to 5 wt.%, forming electrolytes denoted as E‐PFPN and E‐DFEPN, respectively.

Figure 2d, along with Video S1 (G2), S2 (E‐PFPN), and S3 (E‐DFEPN) (Supporting Information), demonstrates that the addition of PFPN and DFEPN effectively suppresses the flammability of the G2 electrolyte; when the ignition source is removed, both E‐DFEPN and E‐PFPN electrolytes are extinguished immediately and fail to burn continuously. The result (Figure S20, Supporting Information) of accelerating rate calorimetry (ARC) test comprehensively validates that DFEPN modifications not only enhance flame retardancy but crucially improve intrinsic thermal safety through multiple protection mechanisms: delayed onset of exothermic reactions and reduced heat release rates. Notably, under both 5 and 10 kW·m^−2^ radiative heat flux exposures, E‐DFEPN demonstrates stable ignition characteristics with nearly identical onset combustion times, showing outstanding flame retardant stability against different thermal intensities. (Figure S21, Supporting Information).

To evaluate the impact of the DFEPN additive on LMA interfacial stability, Figure 2b shows the CE of Li plating/stripping in Li/Cu half cell cycled at a capacity of 1 mAh·cm^−2^ and a current density of 1 mA·cm^−2^ (the experiment data of Li/Cu half cell is shown in Table S1, Supporting Information). Notably, E‐DFEPN extends the cycle life of G2 from 100 to 200 cycles and significantly raises the average CE of G2 from 84.1% (100th cycle) to 89.6% (200th cycle). In the case of G2, CE gradually declines during the initial 100 cycles due to continuous, irreversible depletion of lithium metal as it reacts with the electrolyte. With the addition of PFPN, the CE improves significantly, averaging 90.2% over the first 100 cycles; however, subsequent cycles reveal pronounced fluctuations and a gradual decline in CE, attributable to the unstable SEI on the lithium metal surface. Eventually, the CE drops to 49.9% after 200 cycles. When PFPN is replaced by DFEPN, the CE gradually increases to 91.6% (140th cycle) after ≈60 cycles as a dense beneficial SEI forms, and in the subsequent cycles, E‐DFEPN maintains a high average CE of 91.8%, underscoring the SEI's stability on the lithium metal surface.

The enhanced compatibility of E‐DFEPN with LMA is further evidenced in the Li/Li symmetrical cell cycling tests shown in Figure 2c. E‐DFEPN can lengthen the cycle life of G2 and E‐PFPN from ≈550 to 650 h.

Figures 2e–g present the surface and cross‐sectional morphologies of lithium deposits on Cu foil after 100 cycles, as observed by scanning electron microscopy (SEM), for G2, E‐PFPN, and E‐DFEPN. In G2, the lithium deposition exhibits a sparse, porous structure with numerous irregular fibrous lithium particles distributed randomly (Figure 2e). This porous layer not only raises safety concerns but also adversely promotes side reactions, contributing to the gradual decline in CE observed in Figure 2b. In the SEI formed by E‐PFPN (Figure 2f), the lithium deposition shows a larger grain size than G2, but remains dendritic, which slightly improves performance during the lithium plating/stripping processes. In contrast, E‐DFEPN presents a significantly improved morphology, with dense and homogeneous lithium deposits (Figure 2g). The resulting SEI layer in E‐DFEPN is the thinnest among the tested electrolytes, measuring merely 49.1 µm. The flat, dendrite‐free Li deposition morphology observed in the E‐DFEPN is crucial for maintaining stable lithium plating/stripping throughout cycling. This morphology aligns with the enhanced cycling stability and high CE achieved with E‐DFEPN (Figure 2b).

**Figure 3:**
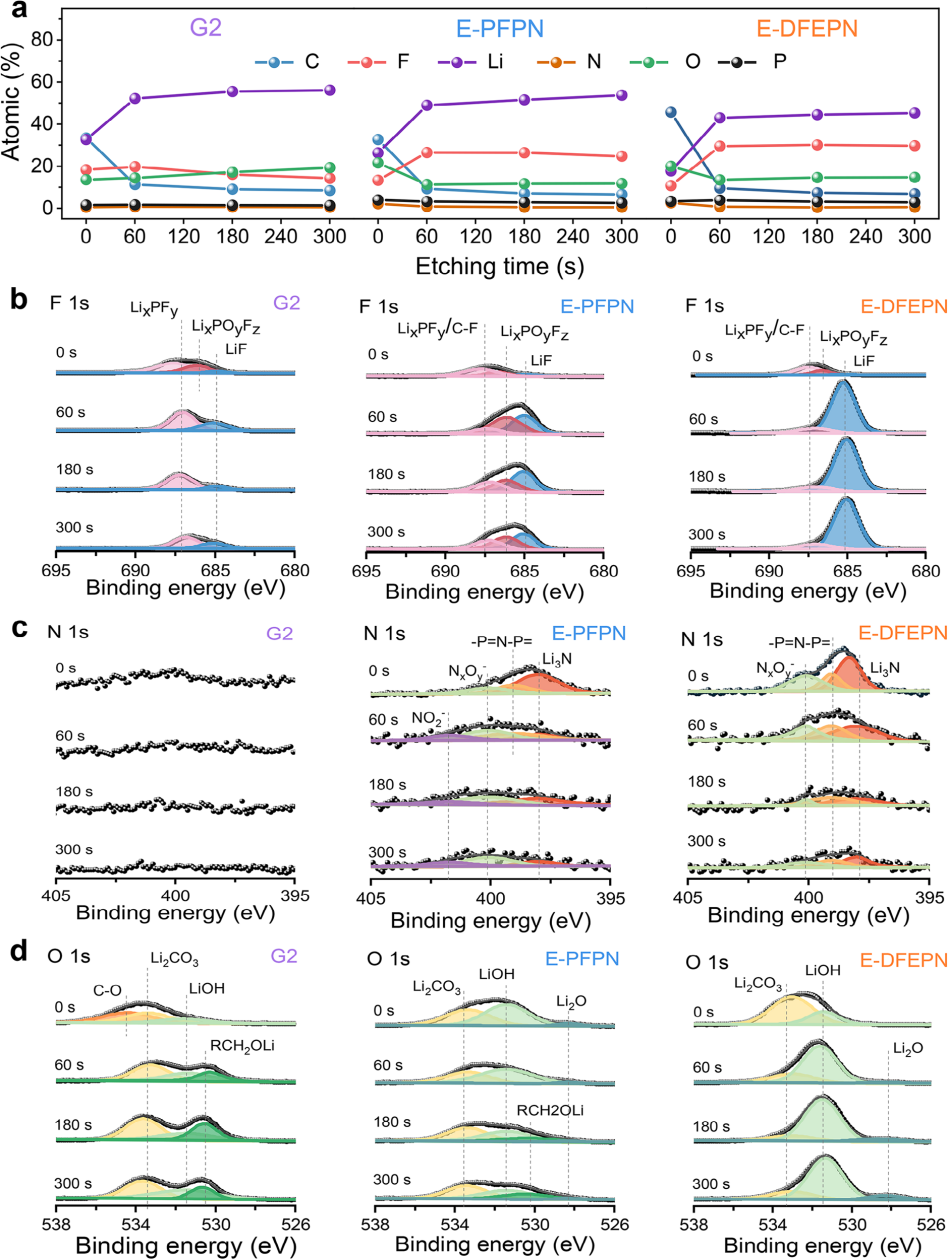
XPS spectra of the SEIs formed by different electrolytes. a) Atomic fractions of the SEI formed at the LMA surface in Li/Cu half cells as a function of etching time in different electrolytes after 100 cycles. b–d) Corresponding XPS spectra of the SEI before and after 60, 180, and 300 s of etching. F 1s XPS spectra (b), N 1s XPS spectra (c), and O 1s XPS spectra (d).

The electrochemical behavior and lithium deposition morphology are closely linked to SEI, prompting the use of X‐ray photoelectron spectroscopy (XPS) to analyze the composition of the SEI formed in different electrolytes. As shown in **Figure** **3**a, a common trend in the SEI compositions from all three electrolytes is that, as etching time proceeds, the ratios of Li, F and O are higher than that of C. This suggests that organic components are primarily located on the SEI surface, whereas inorganic compounds are more concentrated in the interior of the SEI.^[^ ^27^ ^]^ The C 1s spectra (Figure S24, Supporting Information), F 1s spectra (Figure 3b), N 1s spectra (Figure 3c), and O 1s spectra (Figure 3d) further reveal that the organic species in the SEI predominantly consist of C─C/C─H, C─O─C, ROCO_2_Li, and LiCO_3_, which are decomposed products of carbonate solvents. Inorganic species in E‐DFEPN and E‐PFPN include LiF, Li_2_O, and Li_3_N, which arise from decomposition of PF_6_^−^ and phospholipid reagents. In contrast, the inorganic material in G2 is predominantly LiF, formed by decomposition of PF_6_^−^.

In the F 1s spectra (Figure 3b), the atomic content of F in the SEI formed by E‐DFEPN (10.8%) is slightly lower than that of E‐PFPN (13.4%) and G2 (18.3%) before Ar ion sputtering. After 60 s of sputtering, the F content in the SEI formed by E‐DFEPN increases to 29.5%, surpassing that in E‐PFPN (26.5%) and G2 (19.8%). Notably, the LiF content in the SEI formed by E‐DFEPN (89.3%) is significantly higher than that by E‐PFPN (43.5%) and G2 (33.3%), indicating a stronger defluorination effect of DFEPN on the lithium metal surface. A high LiF content in the SEI possesses increased interfacial energy and improved mechanical strength, which promote lateral lithium deposition and suppress dendrite growth.^[^ ^2a,b^ ^]^

The N 1s and O 1s spectra (Figures 3c,d) show that the SEI formed by E‐DFEPN contains higher levels of Li_3_N and Li_2_O than E‐PFPN. This can be attributed to the significant reduction of DFEPN. Li_3_N enhances Li^+^ transport, making SEI an excellent conductor of Li^+^, while Li_2_O helps preventing excessive electrolyte decomposition, contributing to a dendrite‐free lithium deposition morphology. Additionally, the depth profiles of LiF, Li_2_O, and Li_3_N in the SEI formed by E‐DFEPN show their uniform distribution throughout the SEI as the etching time increases. This homogeneous, inorganic‐rich SEI structure, facilitated by DFEPN, offers excellent passivating properties, demonstrating superior compatibility with LMA.

**Figure 4:**
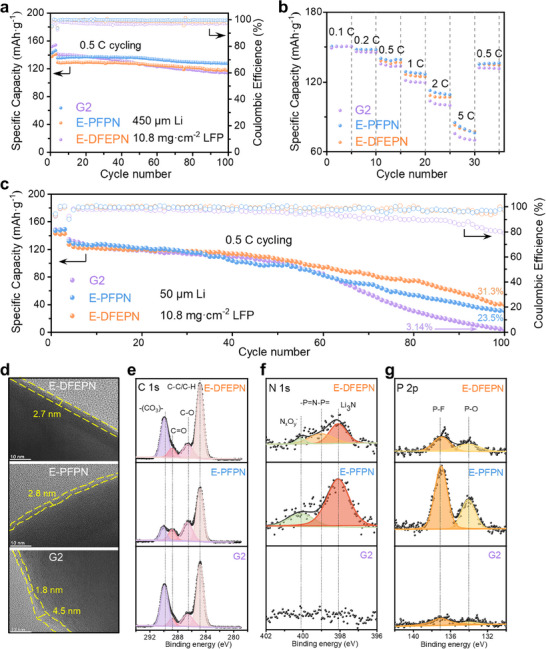
Electrochemical performance of the Li/LFP cell. a) Long‐term cycling performance of Li/LFP half cells with different electrolytes. b) Rate performance of different electrolytes. c) Long‐term cycling performance of Li/LFP full cells between 2.5 and 4.2 V. d) Transmission electron microscopy image of the CEI formed in the LFP cathode after 30 cycles in different electrolytes. e–f) XPS spectra of the CEI with different electrolytes. C 1s spectra (e), N 1s spectra f) and P 2p spectra g).

A Li/LiFePO_4_ (LFP) half cell, featuring a 450 µm Li anode and a high‐loading LFP cathode (10.8 mg·cm^−2^), was assembled and cycled to evaluate the potential use of E‐DFEPN in LMBs. As shown in **Figure** **4**a, both the Li|E‐DFEPN|LFP and Li|E‐PFPN|LFP half cells demonstrate excellent cycling performance, delivering after 200 cycles at 0.5 C a high discharge capacity of 97.5 mAh·g^−1^ (75.5% capacity retention) with an average CE of 98.7% (Figure S25, Supporting Information) and a discharge capacity of 110 mAh·g^−1^ (80.7% capacity retention) with an average CE of 99.5% (Figure S25, Supporting Information), respectively. The voltage profiles of the Li/LFP half cells cycled in E‐DFEPN and E‐PFPN (Figure S26b,c, Supporting Information) show no significant increase in voltage polarization, indicating that DFEPN and PFPN reduce parasitic reactions at the LFP cathode surface. Furthermore, the thin and uniform cathode electrolyte interphase (CEI), with thicknesses of ≈2.7 and 2.8 nm on the LFP surface in E‐DFEPN and E‐PFPN, respectively, suggest that the DFEPN‐ and PFPN‐derived CEI mitigate electrolyte decomposition (Figure 4d), thereby contributing to the remarkable cycling stability of the LFP cathode. In contrast, G2 exhibits a higher overpotential (Figure S26a, Supporting Information) and faster capacity decay (Figure 4a), resulting in lower capacity retention (49.6%) and average CE (96.8%) (Figure S25, Supporting Information) after 200 cycles. Moreover, the CEI formed in G2 is inhomogeneous, with varying thicknesses from 1.8 to 4.5 nm (Figure 4d), which likely increases impedance and accelerates cathode degradation. The rate capability of the Li/LFP half cell with E‐DFEPN mirrors that of E‐PFPN (Figure 4b), with both showing higher specific discharge capacity than that of G2 at various current densities. These results strongly support the outstanding cycling stability of E‐DFEPN in LFP cathode.

To further explore the chemical composition of the DFEPN‐derived CEI, XPS was performed on LFP electrodes after 30 charge/discharge cycles. Compared with the weak N 1s and P 2p signals in the CEI formed by G2, relatively higher N 1s and P 2p peaks are observed in the CEI formed by E‐DFEPN and E‐PFPN (Figures 4f,g), indicating that both DFEPN and PFPN contribute to the formation of the CEI. The appearance of ─P═N─P═(399.0 eV) and Li_3_N (398.1 eV) in the N 1s spectra (Figure 4f) further confirms the involvement of DFEPN and PFPN in the CEI formation process. Given the structural similarities between DFEPN and PFPN, we conclude that the DFEPN or its derivatives undergo ring‐opening polymerization at the LFP cathode surface to form polyphosphazene‐based components and Li_3_N in the CEI, similar to PFPN,^[^ ^28^ ^]^ which can enhance Li^+^ transport and thus, turn the CEI an excellent conductor of Li^+^.^[^ ^29^ ^]^ In the C 1s spectra (Figure 4e), the peaks at 288.8 eV (C═O) and 289.9 eV (‐(CO_3_)‐) can be attributed to the decomposition of DMC. Notably, the intensities of these peaks are lower in E‐DFEPN and E‐PFPN than G2, suggesting that the decomposition of DMC is mitigated in the presence of the DFEPN and PFPN additives. These findings demonstrate that the N‐ and P‐rich CEI generated in E‐DFEPN and E‐PFPN effectively passivates the electrode surface, minimizing carbonate decomposition and enhancing the stability of the LFP cathode.

To further validate the practicality of E‐DFEPN, a Li/LFP full cell with a thin Li anode (50 µm) and a high‐loading LFP cathode (10.8 mg·cm^−2^) was tested between 2.5 and 4.2 V (Figures 4c; Figure S27, Supporting Information). The Li/LFP full cell with E‐DFEPN exhibits exceptional cycling stability, with a high‐capacity retention of 31.3% and an average CE of 98.5% after 100 cycles. In contrast, G2 and E‐PFPN show significantly lower capacity retentions of 3.14% and 23.5%, respectively, after 100 cycles. At 50 °C, the Li/LFP full cell with E‐DFEPN still displays exceptional cycling performance with a capacity retention of 74.9% and average CE of 91.4% after 50 cycles (Figure S22, Supporting Information). The experimental data of Li/LFP half cell and Li/LFP full cell based on different electrolytes are shown in Tables S2 and S3 (Supporting Information), respectively. Compared with NMC811, LiCoO_2_(LCO) possesses a more stable structure and good cycling stability,^[^ ^30^ ^]^ therefore, the LCO with a high loading of 12.9 mg·cm^−2^ is also chosen as the cathode in this study to future evaluate the electrochemical stability of E‐DFEPN. Figure S29 (Supporting Information) demonstrates the cycling performance of E‐DFEPN is superior to that of G2 and E‐PFPN both in Li/LCO half cell and Li/LCO full cell. These results implies that DFEPN additive significantly improves the compatibility with layered oxide system.

## 3 Conclusion

This study addresses a critical bottleneck in the commercialization of LMBs by introducing a generative deep learning model capable of simultaneous multiobjective optimization for electrolyte additive design. The limited availability of structure‐performance data has historically hindered advancements in this field. To overcome this challenge, we developed a molecular categorization derivation method, expanding a few hundred single‐property data points into a comprehensive dataset of 70095 multiproperty data points. This enriched dataset served as the foundation for our advanced model, which integrates an asynchronous limited decoder and an adversarial regulation strategy for latent distribution, enabling high‐efficiency generation of structurally diverse and complex molecules.

Our approach led to the discovery of DFEPN, a flame‐retardant additive that forms stable dual interphases on electrodes. DFEPN outperformed the state‐of‐the‐art additive, PFPN, achieving 33% higher capcacity retention in a Li/LFP full cell. Additionally, DFEPN demonstrated enhanced lithium metal anode compatibility, reduced dendrite formation, and improved SEI properties, as evidenced by higher LiF content and homogeneous SEI distribution.

This study provides a robust framework for advancing LMB safety and performance, addressing the longstanding trade‐offs between electrochemical efficiency and nonflammability. The success of our model highlights the transformative potential of generative deep learning in molecular design, particularly in data‐scarce scenarios.

Future research could incorporate real‐time experimental feedback for model optimization, and exploring additional electrolyte properties such as thermal stability, and low‐temperature performance. These efforts could accelerate the development of next‐generation batteries, paving the way for safer and more efficient energy storage solutions in diverse applications, including electric vehicles and grid storage.

## Conflict of Interest

The authors declare no conflicts of interest.

## Supporting information



Supporting Information

Supplementary Table 1

Supplementary Table 2

Supplementary Table 3

Supplementary Video 1

Supplementary Video 2

Supplementary Video 3

## Data Availability

The data that support the findings of this study are available in the supplementary material of this article.
