# Supplementary material for: Multitarget Generate Electrolyte Additive for Lithium Metal Batteries
Source: Adv Mater. 2025 Jun 18;37(34):2502086. doi: 10.1002/adma.202502086 (PMC12392859; doi:10.1002/adma.202502086)
Supplement: Supplementary file 1 — Supporting Information [file ADMA-37-2502086-s005.docx]

Supporting Information

Multitarget Generate Electrolyte Additive for Lithium Metal Batteries

*Xiangyang Liu, Jianchun Chu, Sa Xue, Daquan Wang, Zhuoyang Lu, Meng Zhang, Yongqi Liu, Xin Xu, Yilin Zhang, Jiangang Long, Lingjie Meng, Jiayin Yuan*, Maogang He**

Table of Content

1. ARECVGA model details 3

1.1. The preparation of dataset 3

1.2. The structure of ARECVGA model 4

1.3. Training of ARECVGA 6

1.4. Sampling molecules by ARECVGA 10

1.5. AEGNN 10

1.6. E(n) equivariance of AEGNN 11

1.7. ALD 12

2. Experiment details 15

2.1. Materials 15

2.2. NMR test on the synthesized DFEPN 16

2.3. Physicochemical and electrochemical test 21

2.4. Electrode characterization 22

2.5. Contact angle 23

2.6. Computational details 24

3. Extended discussion 25

3.1. Random sampled results 25

3.2. Generated results of the model 31

3.3. Model Performance 32

3.4. Gradient experiments 33

3.5. Accelerating Rate Calorimetry (ARC) tests 35

3.6. Temperature-dependency of nonflammability 35

3.7. Temperature-dependency of electrochemical performance 36

3.8. Extended figures 37

4. References 40

# ARECVGA model details

## The preparation of dataset

To introduce generative models into the design of electrolyte additives, we propose the following hypotheses: (1) Molecules that share similar key substructures with known solvents will exhibit comparable properties. In other words, the properties of a molecule are largely determined by its key substructures. (2) The overall properties of a molecule are the average of the properties associated with its constituent key substructures. Based on these hypotheses, we aim to predict the performance of potential electrolyte additives by considering five key performance aspects: conductivity, nonflammability, anodic/cathodic stability, and wettability. These aspects are evaluated for a series of common electrolyte solvents, including cyclic esters, linear esters, ethers, sulfones, phosphates, nitriles, and carbamates (**Figure S1**).

To build our dataset, we select 14,019 molecules from the ZINC database that contain fewer than 30 heavy atoms, at least one of the elements F, P, or S, and at least one characteristic functional group. To enhance the dataset, we perform data augmentation 40 times for molecules containing phosphorus (P) atoms and 30 times for molecules containing sulfur (S) atoms. After augmentation, the final dataset consists of 154,649 molecules. This dataset is then used to calculate and predict the hypothesized performance of the selected molecules based on their substructures and key properties.


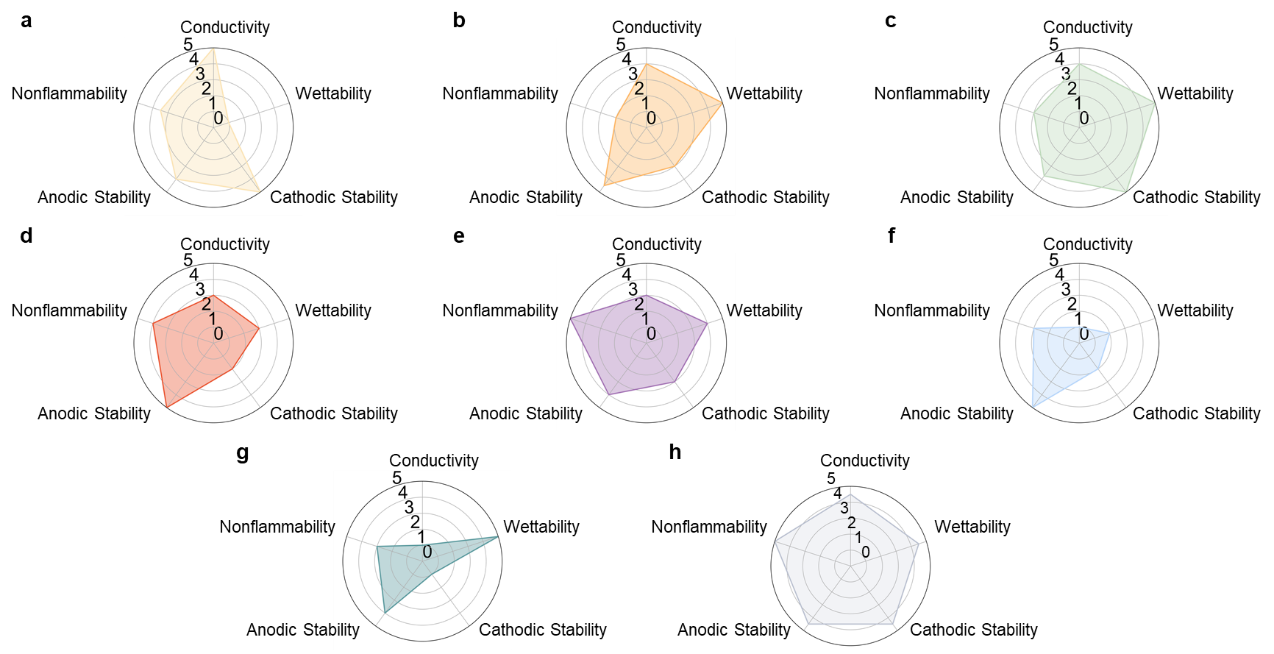


**Figure S1. The performance of common series of electrolyte additives.** **a**, cyclic esters. **b**, linear esters. **c**, ethers. **d**, sulfones. **e**, phosphates. **f**, nitriles. **g**, carbamates. **h**, DFEPN.

Taking the first molecule shown in Figure 1a as an example, which shares the same substructure with ethers and nitriles, we can predict its performance by averaging the performance scores of these two classes of molecules. The performance scores for ethers and nitriles in terms of conductivity, nonflammability, anodic/cathodic stability, and wettability are as follows:

Ethers: [4, 2, 3.75, 5, 5]

Nitriles: [1, 3, 5, 2, 2]

To calculate the predicted performance for the molecule, we take the average of the corresponding performance scores of ethers and nitriles. This results in the following performance values for the molecule:

Conductivity: (4 + 1) / 2 = 2.5

Nonflammability: (2 + 3) / 2 = 2.5

Anodic stability: (3.75 + 5) / 2 = 4.375

Cathodic stability: (5 + 2) / 2 = 3.5

Wettability: (5 + 2) / 2 = 3.5

Therefore, the predicted performance of the molecule is set to [2.5, 2.5, 4.375, 3.5, 3.5], which is the average of the performance scores for ethers and nitriles.

## The structure of ARECVGA model

ARECVGA is an operation on molecular graph: a graph can be represented as, where and follow distribution , is the set of nodes (atoms) in a graph; is the set of edges (bonds) in form *ε_i, j_*=<*v_i_*, *v_j_*> that represents the edge connecting *v_i_* and *v_j_*. The topological structure of can be also represented by a *n*×*n* adjacency matrix A, A*_i, j_* = 1 if there is an edge connecting *v_i_* and *v_j_*, otherwise A*_i, j_* = 0.

**Figure S2**a shows the workflow of the model. Firstly, the node (atom) features *h*, edge (bond) features *e*, adjacency matrix A, node coordinate *x* (Atom coordinates were obtained using experimental-torsion basic knowledge distance geometry provided by RDKit^[1]^), and performance assumption (condition) *c*, are extracted from molecule as shown in Figure S2b, and input to the encoder. Then, the output of encoder will become the latent code by reparametrize process (Figure S2c). Detailly, the output of the encoder is divided into the mean value *m* and variance value *σ* of a Gaussian distribution. In addition, the input of the decoder is a reparametrized value *z* obtained with a random noise *e.* The reparameterization process is shown as:

(S1)

The latent code along with a generated code is judged to be from sample or molecule by discriminator (Figure S2d). To reconstruct the molecule from latent code, firstly, the condition will be repeated *m* times to be in shape [7 (number of performances aspects), *m*] and be concatenated with the latent code, and then the concatenated result is inputted to several MLPs to meet the input form of decoder. Finally, the output of the decoder will resume to molecule by the ALD (Figure S2f).


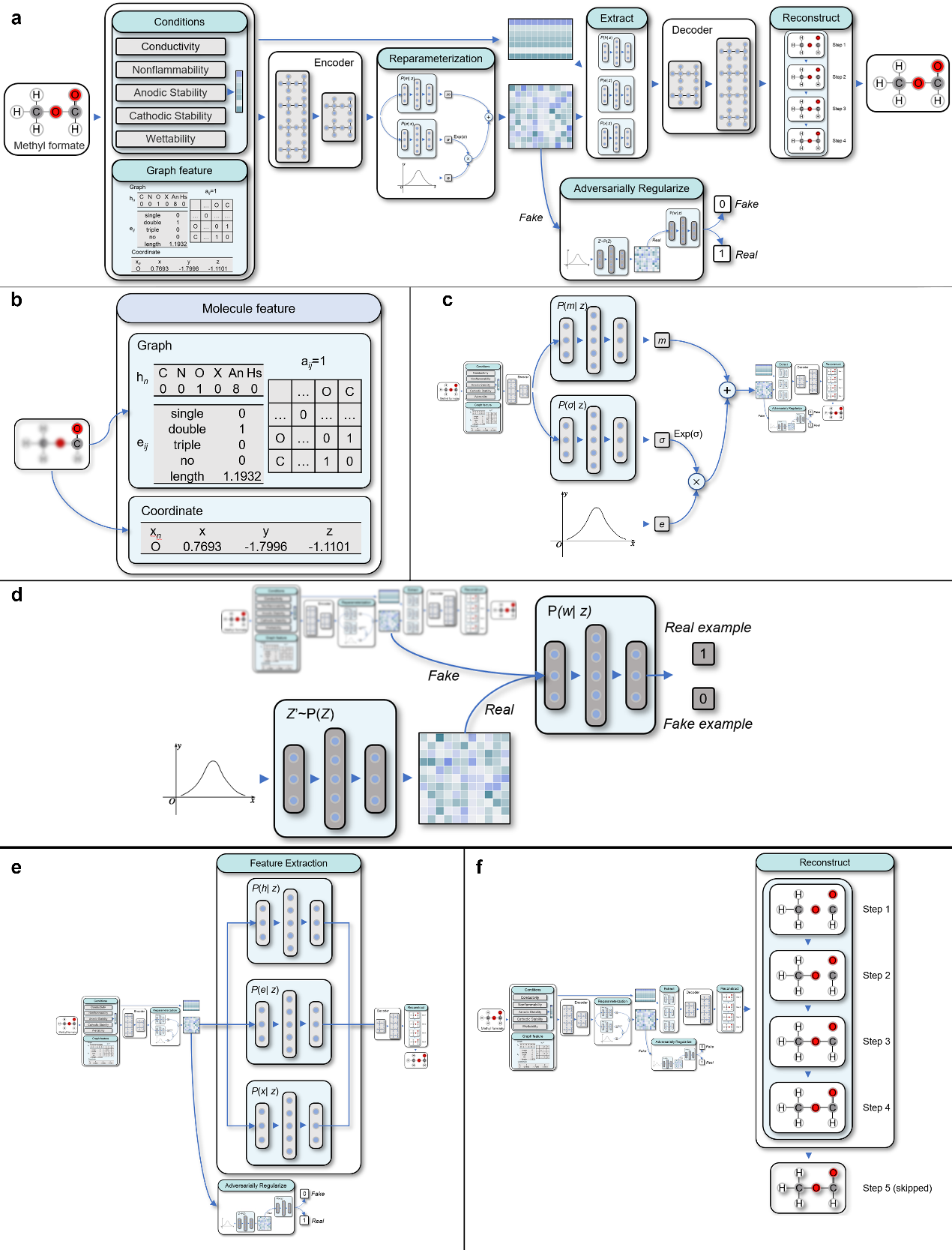


**Figure S2. The workflow and details of ARECVGA.** **a**, The summary of ARECVGA. **b**, The processed graph details of model input. **c**, The reparametrize step. **d**, The generator and discriminator. **e**, The process from latent code to the input of decoder. **f**, Asynchronous limited decoder.

## Training of ARECVGA

Detailly, ARECVGA embeds the nodes in the graph (the node embedding includes its edge information, and the shape of latent code is number of nodes × latent dimension) to latent codes by encoder, decode the latent codes to nodes by decoder and enforces the latent codes follow given distribution by generator and discriminator.

Specifically, is a deterministic encoder function with parameters *,* and is the decoder with parameters to reconstruct *v, ε*. Encoder and decoder should minimize the reconstruction error by training and:

(S2)

Also, the encoder should regularize the latent code distribution with the prior distribution by minimizing Wasserstein distance .

(S3)

In another words, the output of encoder should be as indistinguishable as possible for the discriminator *f_w_* from the results generated by the generator over noise *s* sampled in Gaussian distribution. To aid this goal, the generator also needs to be trained to confuse discriminator:

(S4)

In contrast, the discriminator should try its best to distinguish if a code is output by encoder or generator:

(S5)

The encoder does not output the latent code *z* directly, but output means and variances value. The final *z* is a sample in the Gaussian distribution who’s mean and variance are the output of encoder, as using reparametrizing trick in VAE.

| Algorithm 1 ARECVGA training |
| --- |
| **for** epoch **do**  Train the encoder and decoder , to minimize reconstruction error:  For a graph with *m* nodes , conditions *c*, and edges *ε*   1. get its latent code    1. decode back to    1. backward the reconstruction loss   Train the discriminator (*w*), to regularize the distribution of encode latent code:  let   1. encode the latent codeby encoder, generate latent code    1. backward the generation similarity loss   Train the encoder and generator to confuse discriminator   1. encode the latent codeby encoder, generate latent code    1. backward the discriminator loss   **end for** |

The model is trained for maximum 60 epochs and the model parameters with lowest loss for validation set is chosen for further evaluation. ADAM algorithm is used to optimize the model parameters, learn rate for encoder and decoder is 1e-3, for generator and discriminator is 5e-7, for predictor is 1e-2, the weight decay is 1e-6. And the batch size is 10. Each property is normalized by subtracting the mean and dividing by the variance. For each atom, its input feature has 25 dimensions. 4 dimensions, as a whole, is a one-hot encoder of explicit hydrogen num:. And the other 21 dimensions of the input feature, as a whole, is an atomic number mixed one-hot vector, which expands each atom (X indicates ‘not an atom’) into 3 dimensions according to their atomic number, so that finally this vector has 7 atom types × 3 expanding times + 4 dimensions for explicit hydrogen num one-hot = 25 dimensions.

To capture nonlinear relationships, we expand each number in the atom one-hot encoding three times:

(S6)

where *α_t_* is the number in the one-hot (1 or 0), *α_n_* is the atomic number, and *c* = 32 is a hyperparameter to limit the range of *d_i_* (determined by the atomic weight of sulfur). The expanded one-hot encoding is concatenated to the original node feature vector *v*. For example, because the electrolyte may include atoms in, the atom type one-hot is 7 dimensional. Therefore, the atom feature has 7 atom types × 3 expanding times = 21 dimensions. Detailly, for a carbon atom, its origin atom one-hot is [1, 0, 0, 0, 0, 0, 0], where ‘1’ demonstrates this atom is a carbon, ‘0’ demonstrates this atom is not other atoms. In contrast, the one-hot of a fluorine atom is [0, 0, 0, 1, 0, 0, 0]. After expanding, the carbon one-hot [1, 0, 0, 0, 0, 0, 0] will be an array with 21 numbers, in which the first 3 numbers represent the carbon together, and the 10^th^-12^th^ numbers represent the fluorine together. The first 3 numbers are calculated as below:

(S7)

As for other numbers, because other numbers of the one-hot are 0 (*α_t_=0*), the array *d* for these numbers will be [0, 0, 0]. And finally, the atom feature will be transformed from [1, 0] to [1, 0.1875, 1.125, 0, 0, 0, 0, 0, 0, 0, 0, 0, 0, 0, 0, 0, 0, 0, 0, 0, 0].

The edge feature has 5 dimensions in which 4 dimensions are for bond type one-hot and a dimension for bond length. Table 2 is the structure of the model. The hidden feature of nodes *h* has 40 dimensions for ZINC, the latent code *z* has 12 dimensions for each atom. The structure of the model is shown in **Table S1**, in which FC means full connect layer, is node features, *z* is latent code and is the latent code from generator.

**Table S1. Model structure and layer forms**

| Part | Layer and (Layer form) | Input | Output |
| --- | --- | --- | --- |
| Encoder  ()  | Node embedding (FC) |  | *h*^(0)^ |
|  | 1^st^ Graph embedding (AEGNN) | *h*^(0)^, *e*^(0)^, *x*^(0)^ | *h*^(1)^, *e*^(1)^, *x*^(1)^ |
|  | 2^nd^ Graph embedding (AEGNN) | *h*^(1)^, *e*^(1)^, *x*^(1)^ | *h*^(2)^, *e*^(2)^, *x*^(2)^ |
|  | 3^rd^ Graph embedding (AEGNN) | *h*^(2)^, *e*^(2)^, *x*^(2)^ | *h*^(3)^, *e*^(3)^, *x*^(3)^ |
|  | Mean (FC) | *h*^(3)^,conditions | mean |
|  | Variance (FC) | *h*^(3)^,conditions | variance |
|  | Reparameterization | mean, variance | *z* |
| Decoder  ()  | Atom num MLP (FC) | *z*, conditions | Atom num |
|  | Z embedding MLP (FC) | *z*, conditions | *h**^(0)^ |
|  | Atom position MLP (FC) | *z*, conditions | *x**^(0)^ |
|  | Edge embedding MLP (FC) | *z*, conditions | *e**^(0)^ |
|  | 1^st^ Graph embedding* (AEGNN) | *h**^(0)^, *e**^(0)^, *x**^(0)^ | *h**^(1)^, *e**^(1)^, *x**^(1)^ |
|  | 2^nd^ Graph embedding* (AEGNN) | *h**^(1)^, *e**^(1)^, *x**^(1)^ | *h**^(2)^, *e**^(2)^, *x**^(2)^ |
|  | 3^rd^ Graph embedding* (AEGNN) | *h**^(2)^, *e**^(2)^, *x**^(2)^ | *h**^(3)^, *e**^(3)^, *x**^(3)^ |
|  | Node embedding (FC) | *h**^(3)^ |  |
| Generator  ()  | (FC) | Sampled code |  |
| Discriminator  (*w*) | (FC) | ,  | 0 ~ 1 |

## Sampling molecules by ARECVGA

To sample a molecule, a sample from prior distribution is fed to generator firstly (**Figure S**3). After then, the generated latent tensor and conditions are fed into decoder for decoding. And finally, the ALD is used to transfer the output of decoder to molecular structure.


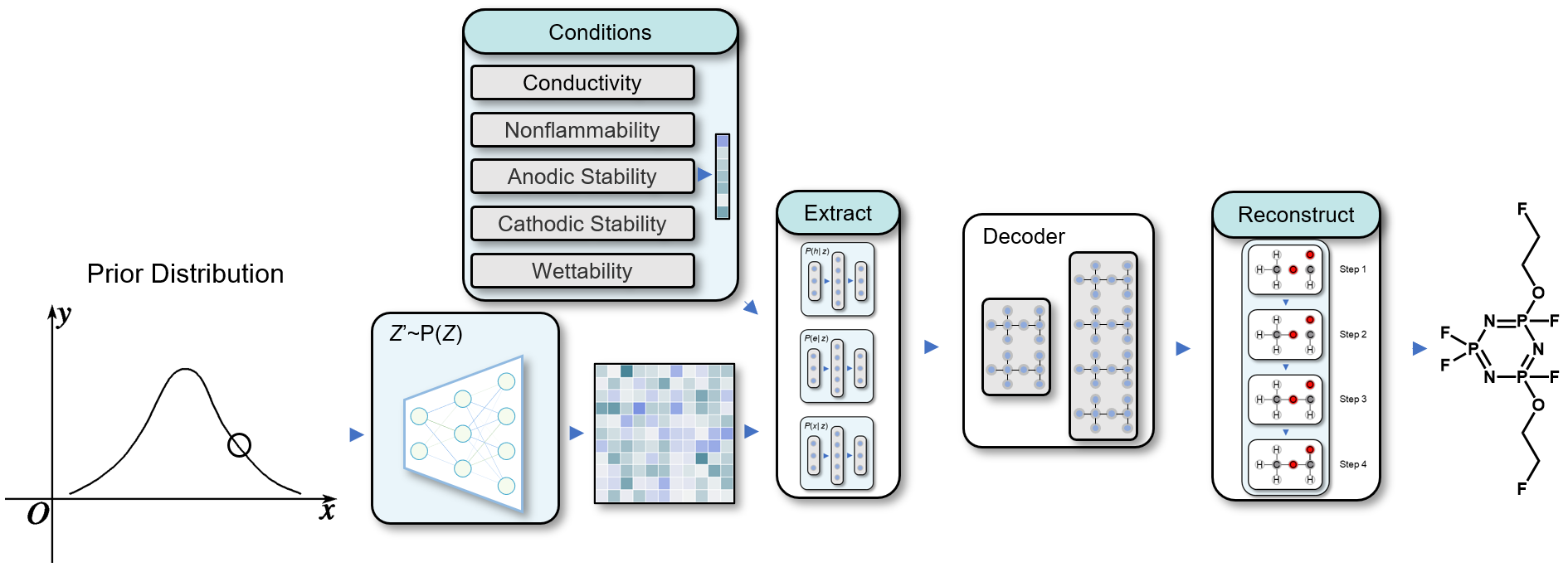


**Figure S3. The sampling process of ARECVGA.**

## AEGNN

Each layer in encoder and decoder of ARECVGA uses AEGNN for propagation. the AEGNN model enhances the summarization step with an attention mechanism to calculate edge weights, improving the message-passing process. For the node (atom) v*_i_* in *l*^th^ layer of encoder and decoder, which has a coordinate in physical space (node coordinate), the output node representation *h_i_^l+^*^1^ and new coordinate is calculated by a combination of the node *v_i_* itself, neighbor edge *e_ij_***,** neighbor node *v_j_*, the Euclidean distance and the adjacency matrix. Specifically, we define an edge operation, coordinate operation, node operation , the edge information from the neighbor m*_ij_* and the calculation is shown from Equation(S8) to Equation(S11).

(S8)

(S9)

(S10)

(S11)

are the neighbor nodes of *v_i_*, *m_ij_* is the message from *h_j_^l^* and to *h_i_^l^*, and *m_i_* is an aggregated message to *h_i_^l^*. The parameter in Equation(S9) is a parameter to normalize the influence of neighbors to avoid nodes with many neighbors losing information about themselves. For the input of first layer in encoder and the output of last layer in decoder, and , respectively. If the *l*^th^ layer is the last layer of encoder, . For encoder, we do not renew the coordinate by Equation(S9). Subsequently, the edge operation , coordinate operation and node operation can be MLP models or attention model. In this work, node operations, edge operations, and the attention model are MLP models. Each MLP contains an input layer, hidden layers, and an output layer. The number of neurons in the input layer matches the number of input features. The input layer gets the input values and sends them to the first hidden layer. Then the first hidden layer processes the values and outputs a value to the next layer. The value of *i*^th^ neuron in (*k+*1)^th^ layer *z_i_^k+^*^1^ is calculated as:

(S12)

where *w_ij_^k^*^+1^ is the weight of *j*^th^ neuron in (*k*+1)^th^ layer, *b^k^*^+1^ is the bias of (*k*+1)^th^ layer, and 𝜎 is an activation function.

## E(n) equivariance of AEGNN

E(n) equivariance ensures that the model output remains consistent under translations, rotations, and reflections. For a translation vector, and an orthogonal matrix , the model must satisfy:

(S13)

For translation, we apparently have

(S14)

For rotation and reflection, we have

(S15)

Therefore, the translation, rotation, and reflection operations do not change , Similarly, we can deduce that the message passed by edges is invariant:

(S16)

## ALD

The interpretation of model output is divided into 2 technical routes: synchronous and asynchronous constructing. Normally, synchronous constructing determines every atom and bond at the same time, which is to find the maximum likelihood result for every position (for SMILES VAE, a position is a SMILES character, for graph VAE, a position is an item in node and edge array), as

(S17)

Whereas it is necessary to decode asynchronously to constrain the decoding result to follow the chemical rules as grammar VAE, SDVAE, etc. The asynchronous decoding is generally to generate atoms and bonds one by one in the way of spanning tree. If the sampling is not correct at one step, the development of the spanning tree will be led to a completely different path. The gains outweigh the losses if we miss the molecules that the model intends to output only to boost the output validity. A proper approach is which can restore the molecular structure represented by the probability distribution as much as possible, while ensuring the validity of the model output. This approach can be regard as a complex version of work assignment problem, but even simplifying the problem by pruning, the computational cost is still extremely high. In this work, we consider the sampling process as a work assignment problem, but solve it by combining the core idea of synchronous and asynchronous sampling. The sampling process is shown below:

Hypothesis 1: All the atom types and the num of their implicit hydrogens are correct.

Hypothesis 2: During reconstruction, the incorrect position mostly has higher cross entropy.

Step 1: Sample all the atoms and their implicit hydrogens, determine their max valence by Algorithm 2, then according to their max valence. Delete all the impossible bond sampling branches.

Step 2: Sample a bond for the atoms whose remain valences are 1.

Step 3: Sample a bond for the atoms (such as oxygen) whose max valence is 2 and have been sampled a bond during step 2.

Step 4: Sample a bond for the atoms that have not connected to any other atoms yet.

Step 5: Sample bonds for the atoms that have not reached max valence.

During sampling, check the target atom remain valence and if there is any contradiction, according to Hypothesis 2, sample the bond with second highest possibility. After step 1, every step starts with checking if the molecular graph has been complete.

Briefly, we firstly determine every heavy atom and their implicit hydrogens, then sample the bonds under the constraints that the atoms should not be linked to more bonds than its limitation, and the molecular graph should be a connected graph.

To solve the problem that some atoms have not only 1 common valence (such as trivalent phosphorus and pentavalent phosphorus), we determine the max valence of each atom when they are sampled. For an atom, this determination firstly samples bonds for every possible maximum valence situation, then choose the maximum valence with lowest probability loss:

| Algorithm 2 Determine the max valence for atom |
| --- |
| \| **Input:**  *V* : decoded node probability matrix  *E*: decoded edge probability matrix  *L*: Atom list  *A*: Adjacency matrix  *a*: An instance of the ***Class*** Atom with attribute *m* (int or list)  **Output:**  *a*: An instance of the ***Class*** Atom with attribute *m* (int)  **Create:**  *V_copy*: a copy of decoded node probability matrix  *E_copy*: a copy of decoded edge probability matrix  *A_copy*: a copy of Adjacency matrix  *PL*: a list whose length is equal to the length of *a.m*, created if *a.m* is a list // probability loss  **if** *a*.*m* is a list **do**:   \| **for** *i* in range of *a.m* length **do**:   \| *max_valence* ← *a.m*[*i*]  *valence* ← *a.c*  **if** *a.c* > *max_valence* **do**:   \| *PL*[*i*] ← 100000 // an extreme large number  **continue** \| \| --- \|   **else:**   \| *SP* ← a list full of 0, whose length is number of atoms // sampled probability  *OP* ← *E_copy*[the position of *a* in *L*] // output probability  **repeat**   \| Sample maximum likelihood edge argmax(*E_copy*[*i*])[0] with bond type  *valence* += argmax(*E_copy*[*i*, argmax(*E_copy*[*i*])[0]]) + 1  *A_copy*[i, argmax(*E_copy* [*i*])[1]] ← 1  *E_copy*[argmax(*E_copy* [*i*])[0]] ← 0  *SP*[argmax(*E_copy* [*i*]] ← 1 \| \| --- \|   **until** *valence == max_valence*  *PL*[*i*] ← average(abs(*SP* - *OP*)) \| \| --- \| --- \| \| \| --- \| --- \| --- \| --- \|   *a.m* ← *a.m*[argmin(*PL*)] \| \| --- \| --- \| --- \| --- \| --- \| \| \| --- \| --- \| --- \| --- \| --- \| --- \| |
| **return** *a* |

The comparison between asynchronous decoding, synchronous decoding and ALD are shown in **Figure S4**, which is a detailed version of Figure 1c. The figure also shows the common errors occur when using synchronous or asynchronous decoding.


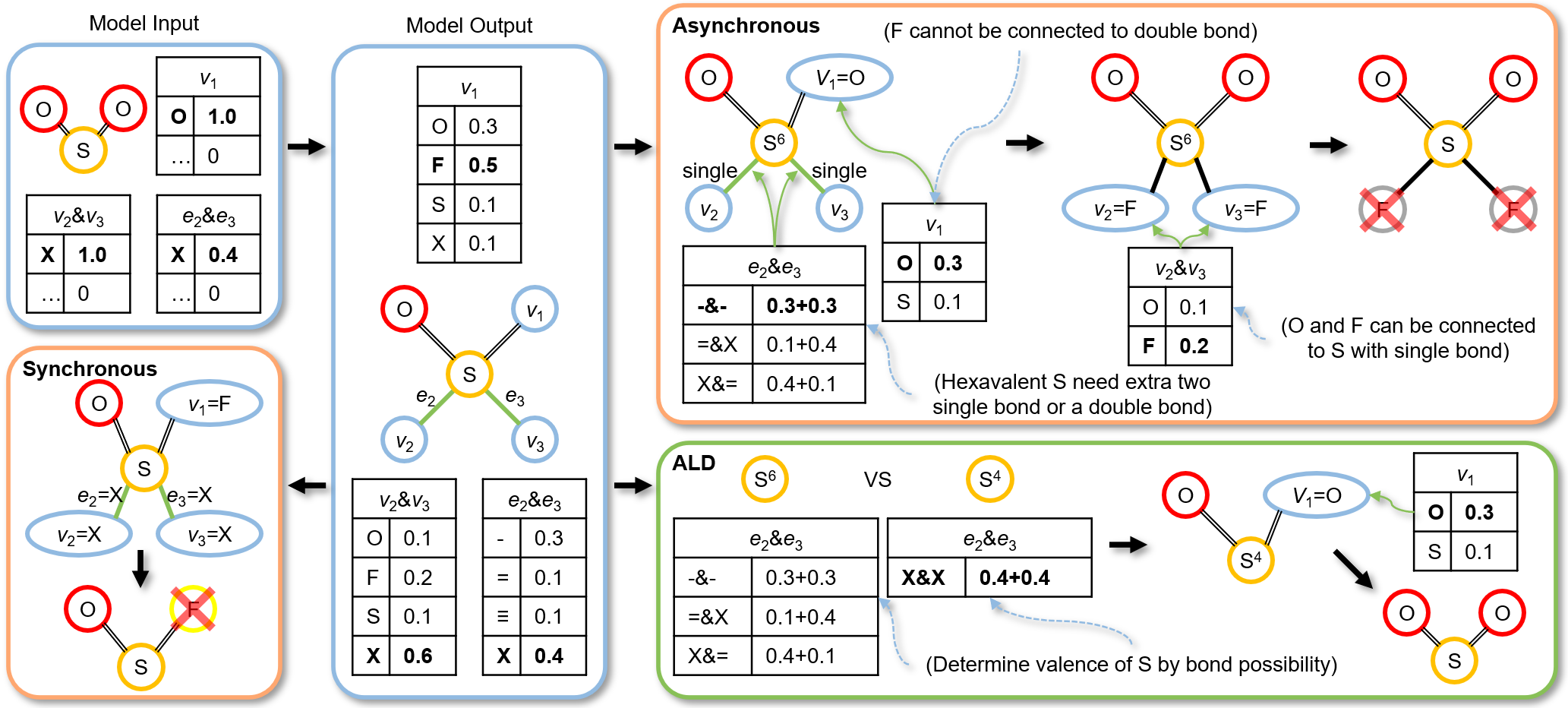


**Figure S4. The comparison between asynchronous decoding, synchronous decoding and ALD**

# Experiment details

## Materials

The materials for performance evaluation: The blank electrolyte with 1.0 M LiPF_6_ in EC/DMC (1:1 by vol) and ethoxy(pentafluoro)cyclotriphosphazene (PFPN, > 98.0%) were purchased from Dodo Chem Co. and Aladdin, respectively. PFPN was dried with 4 Å molecular sieve for 72 h before use. The coin batteries (CR 2032) with polypropylene membrane (Celgard 3501, Suzhou Sinero Technology Co., LTD) were employed to assemble Li/Cu, Li/Li and Li/LiFePO_4_ (LFP) batteries. Lithium with thickness of 450 μm and 50 μm was supplied by Dodo Chem Co. Ltd. and Guangdong Canrd New Energy Technology Co. Ltd., respectively. LFP cathode with an active material loading of 10.8 mg·cm^-2^ and areal capacity of 1.62 mAh·cm^-2^ was provided by Guangdong Canrd New Energy Technology Co. Ltd. The mass percent of active material (LFP), conductive additive and binder on electrode is 91.5%, 3.5%, 5%, respectively. Polypropylene membrane (Celgard 3501) with the thickness of 25 μm was obtained from Suzhou Sinero Technology Co. LTD. Copper foil with the thickness of 23 μm was obtained from Shenzhen Huaqing Materials Technology Co. LTD. The polypropylene membrane and LFP cathode were dried at 50 ˚C and 105 ˚C for 4 h, respectively. All the electrolytes and coin cells were prepared in an Ar-filled glovebox (O_2_ < 0.1 ppm, H_2_O < 0.1 ppm). Each coin cell uses 75 μL of electrolyte. The mass of all components of the battery is shown in **Table S2**.

**Table S2 Mass of all components of the battery**

| Compents | Mass/ g |
| --- | --- |
| Negative terminal | 0.8653 |
| Positive terminal | 0.8959 |
| Disk spring | 0.3639 |
| Stainless steel spacer | 1.4433 |
| Separator | 0.0036 |

The materials for DFEPN synthesizing: hexafluorocyclotriphosphazene (N_3_P_3_F_6_, HFCP, ≥ 97.0%, Adamas Reagent Co.Ltd., CAS 15599-91-4), 2-fluoroethanol (FCH_2_CH_2_OH, 2-FE, ≥ 98%, CAS 371-62-0), triethylamine and anhydrous potassium carbonate phosphate purchased from Adamas Reagent Co. Ltd.

Synthesis of DFEPN: Acetonitrile (100 mL), hexafluorocyclotriphosphazene (10 g, 40.17 mmol), and 2.57 g of 2-fluoroethanol (40.17 mmol) were sequentially added to a single-necked flask, followed by the addition of 5.56 g of anhydrous potassium carbonate (40.2 mmol) under magnetic stirring at 600 rpm. The reaction continued at ambient temperature for 24 hours. After completion, centrifugation at 10,000 rpm for 10 minutes yielded separate precipitate and supernatant fractions. The precipitate was washed with 50 mL of acetonitrile. Combining the two supernatants and removed the most solvent and unreacted HFCP (easy to sublimation, b.p. 50.9 °C), the resulting mixture was added dropwise to 2 L of deionized water and allowed to stand for 48 hours to remove unreacted 2-fluoroethanol. The bottom layer, consisting of colorless liquid, was dried with anhydrous sodium sulphate (Na_2_SO_4_), collected and obtained as DFEPN.

(S18)

## NMR test on the synthesized DFEPN

**Figures S5–S7** show the nuclear magnetic resonance spectroscopy (NMR) spectra (^1^H, ^13^C, ^19^F, and ^31^P) of the synthesized DFEPN. In the ^1^H-NMR spectrum (**Figure S8**), the peak at 3.92 ppm, which corresponds to the O-H group in 2-fluoroethanol, disappears in DFEPN, confirming that the O-H group has been removed upon reaction. The four peaks attributed to the C-H remain, but their chemical shifts changed, indicating successful reaction between 2-fluoroethanol and HFCP. In the ^13^C-NMR spectrum (**Figure S9**), the carbon signals for 2-fluoroethanol appear as four distinct peaks due to presence of fluorine atom. In DFEPN, the substitution of two 2-fluoroethoxy groups on the two sides of the cyclotriphosphazene ring results in a shift of these four peaks. Additionally, two groups of multiple peaks appear near 82.3 and 80.6 ppm, and two doublet pairs are observed at 67.54 and 66.99 ppm (**Figure S10**). This confirms the successful grafting of two 2-fluoroethoxy groups onto separate phosphorus atoms of the cyclotriphosphazene, rather than onto a single phosphorus atom, verifying the successful synthesis of DFEPN. In the ^19^F-NMR and ^31^P-NMR spectrum (**Figure S11 and S12**), the splitting patterns are too complex for detailed analysis. However, the ^19^F-NMR spectrum clearly shows the shift from two groups of symmetric multiple peaks in HFCP (corresponding to the six fluorine atoms on either side of the cyclotriphosphazene ring) to four groups of distinct multiple peaks in DFEPN, indicating the successful introduction of the 2-fluoroethoxy groups. **Figure S13** displays the high-resolution mass spectrum of DFEPN. These results collectively confirm the successful synthesis of DFEPN.


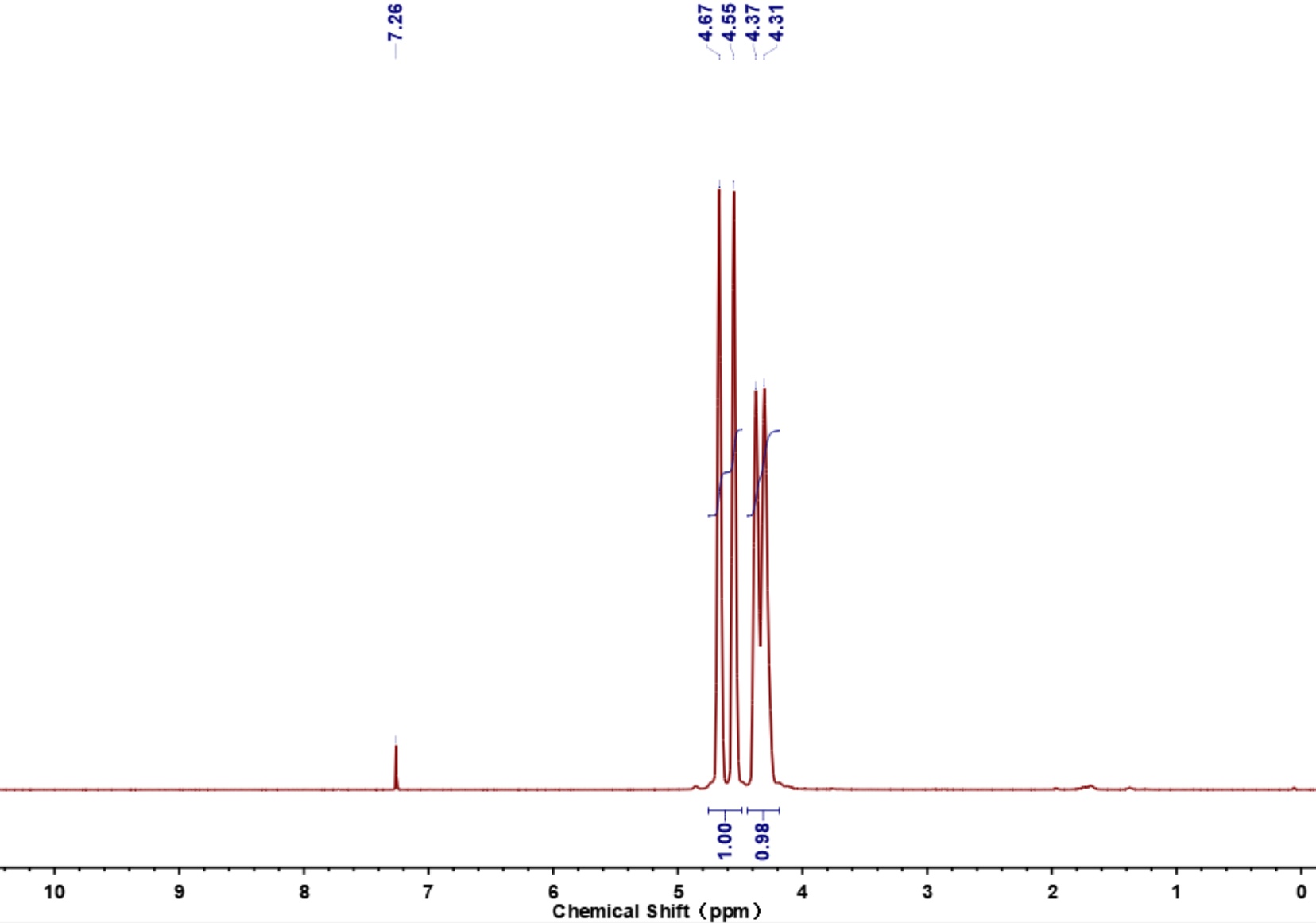


**Figure S5. ^1^H-NMR spectrum of DFEPN.**


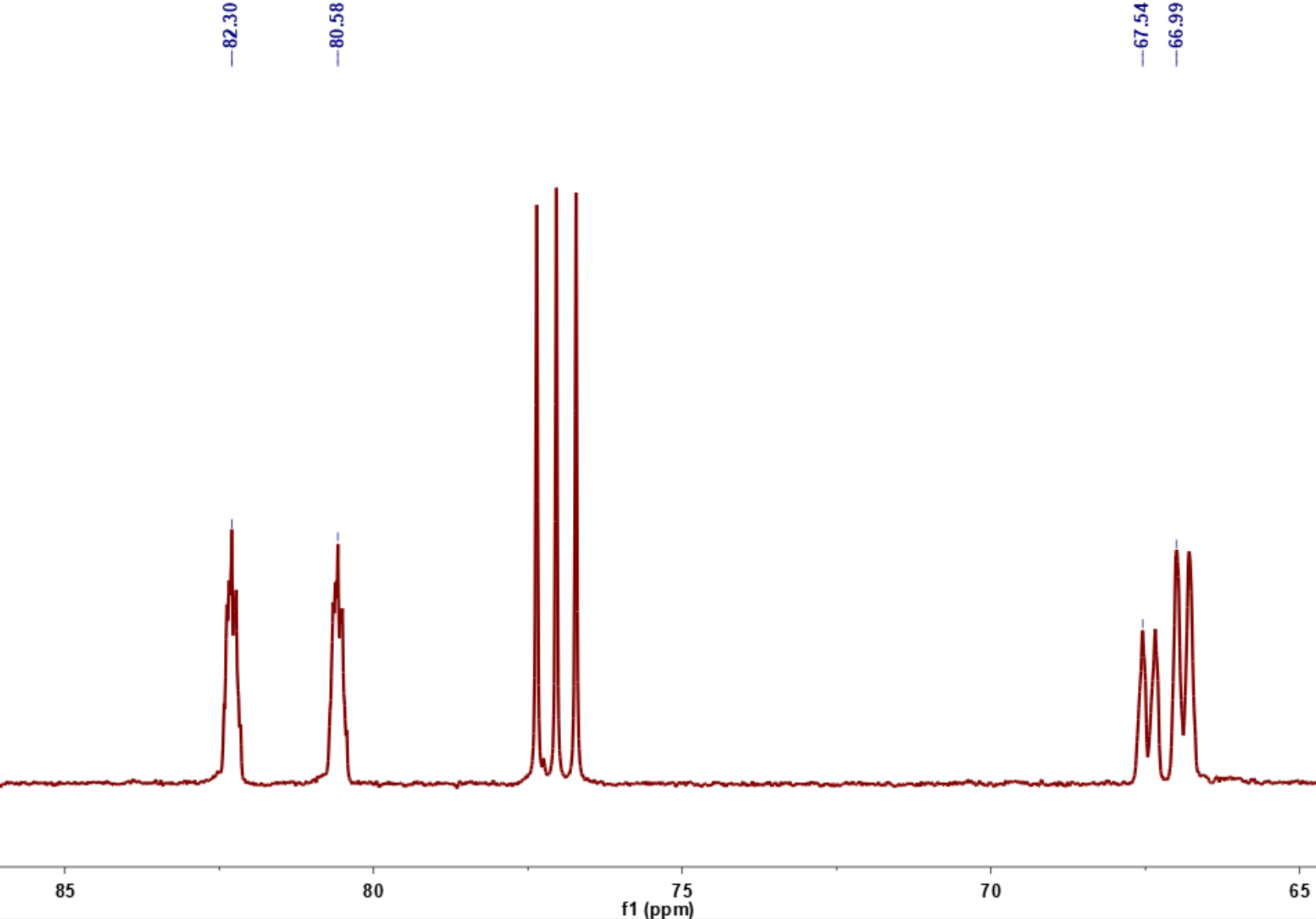


**Figure S6. ^13^C-NMR spectrum of DFEPN.**


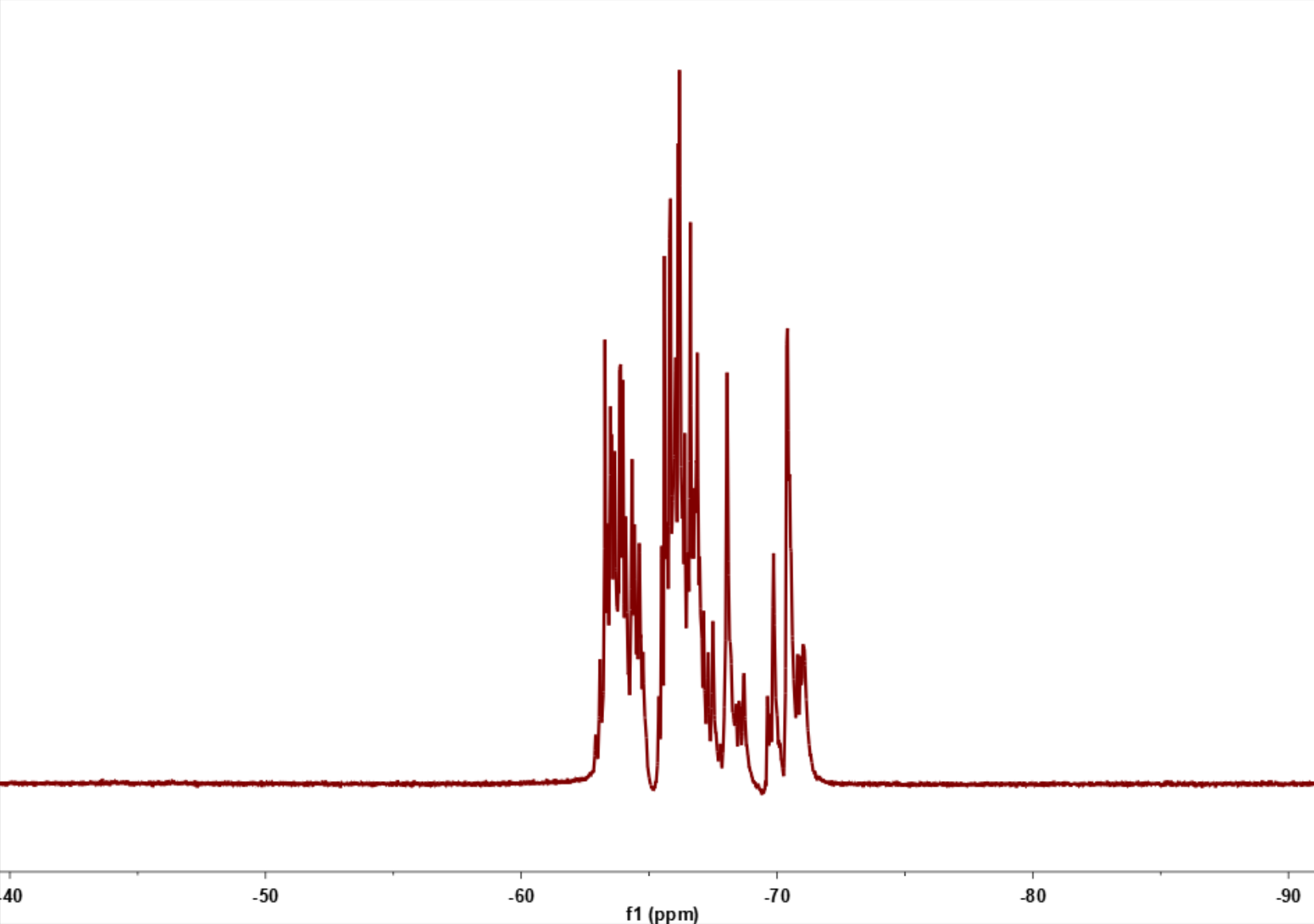


**Figure S7. ^19^F-NMR spectrum of DFEPN.**


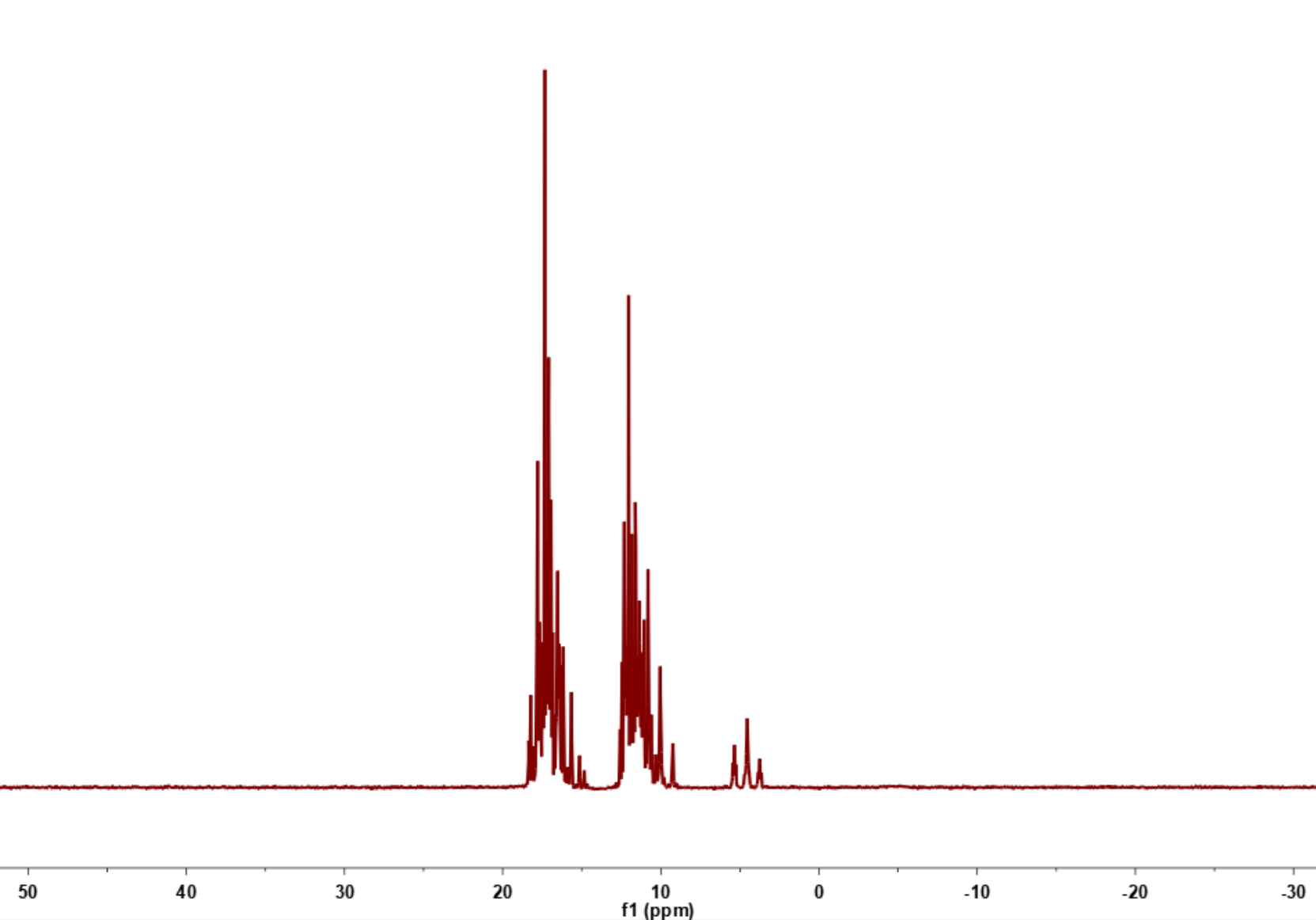


**Figure S8. ^31^P-NMR spectrum of DFEPN.**


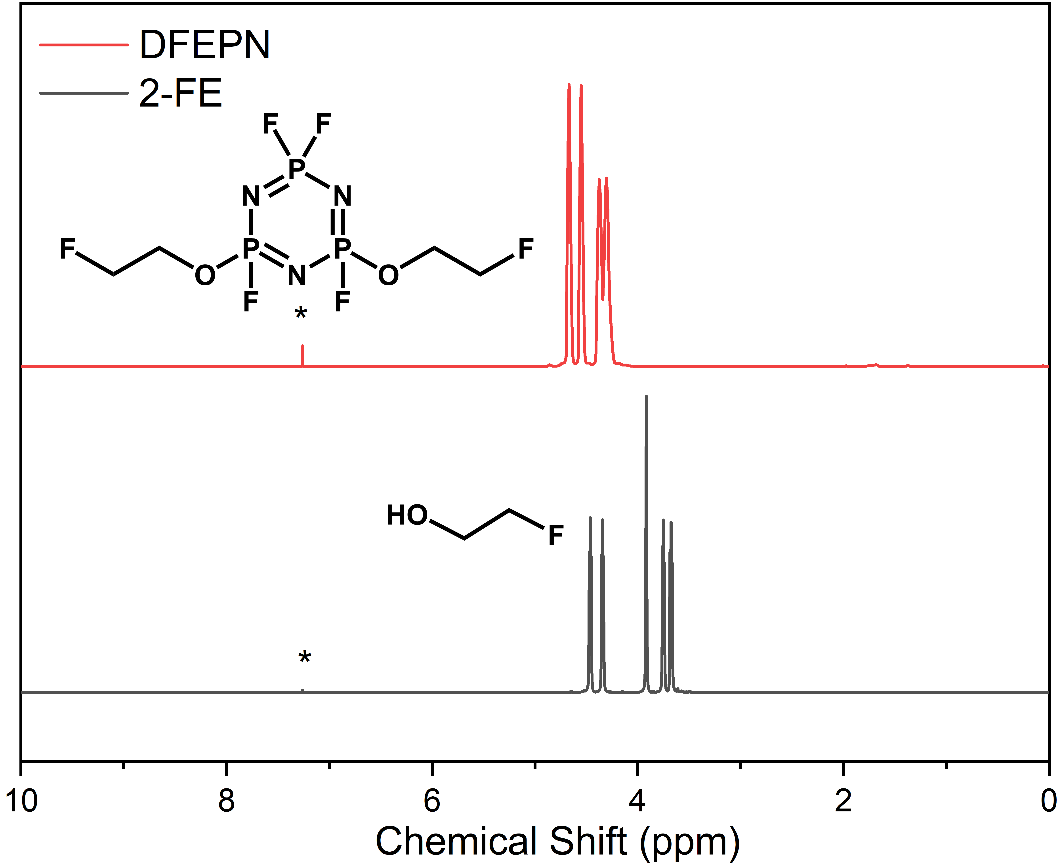


**Figure S9. ^1^H-NMR spectrum of synthesized DFEPN *vs.* 2-fluoroethanol.**


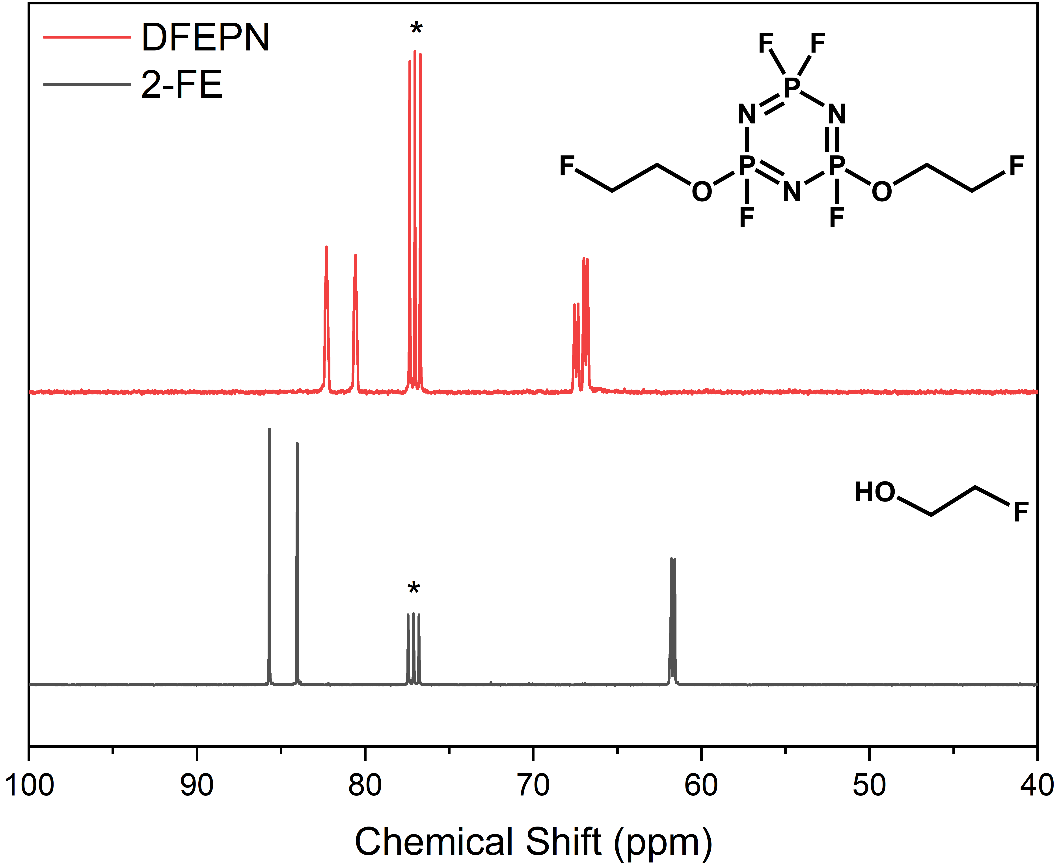


**Figure S10. ^13^C-NMR spectrum of synthesized DFEPN *vs.* 2-fluoroethanol.**


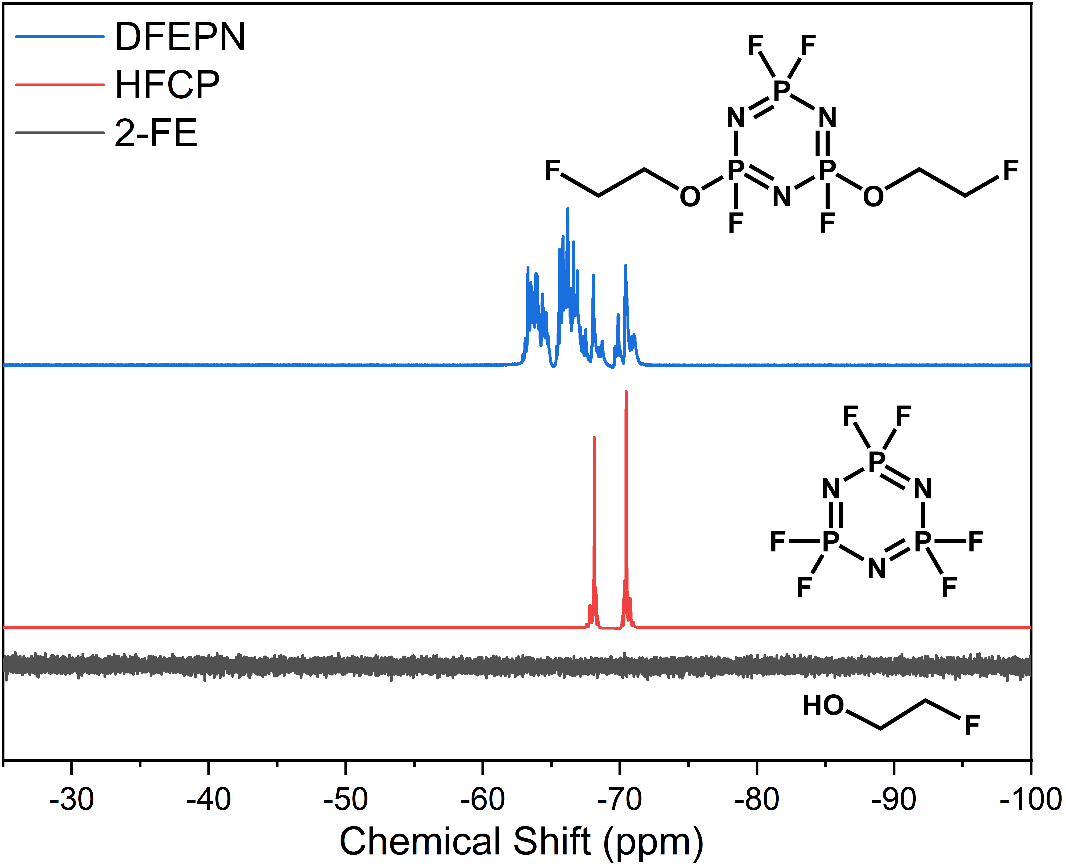


**Figure S11. ^19^F-NMR spectrum of synthesized DFEPN *vs.* HFCP.**


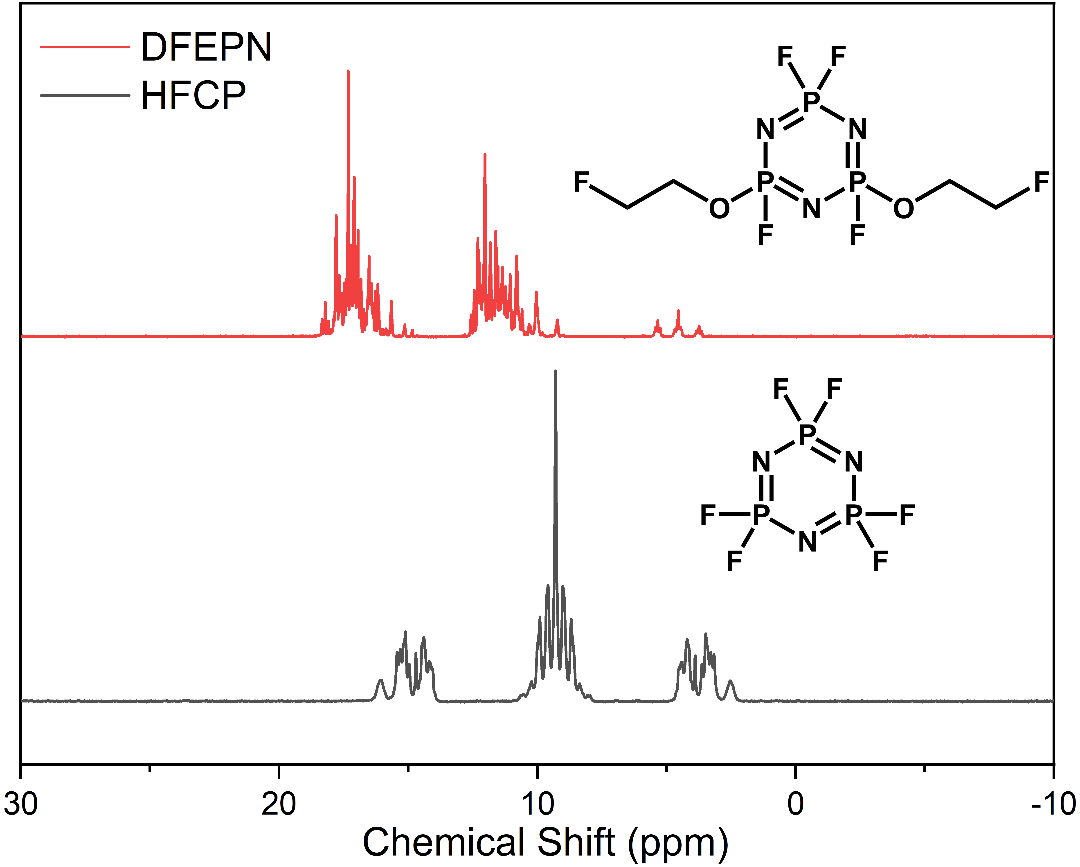


**Figure S12. ^31^P-NMR spectrum of synthesized DFEPN *vs.* HFCP and 2-fluoroethanol.**


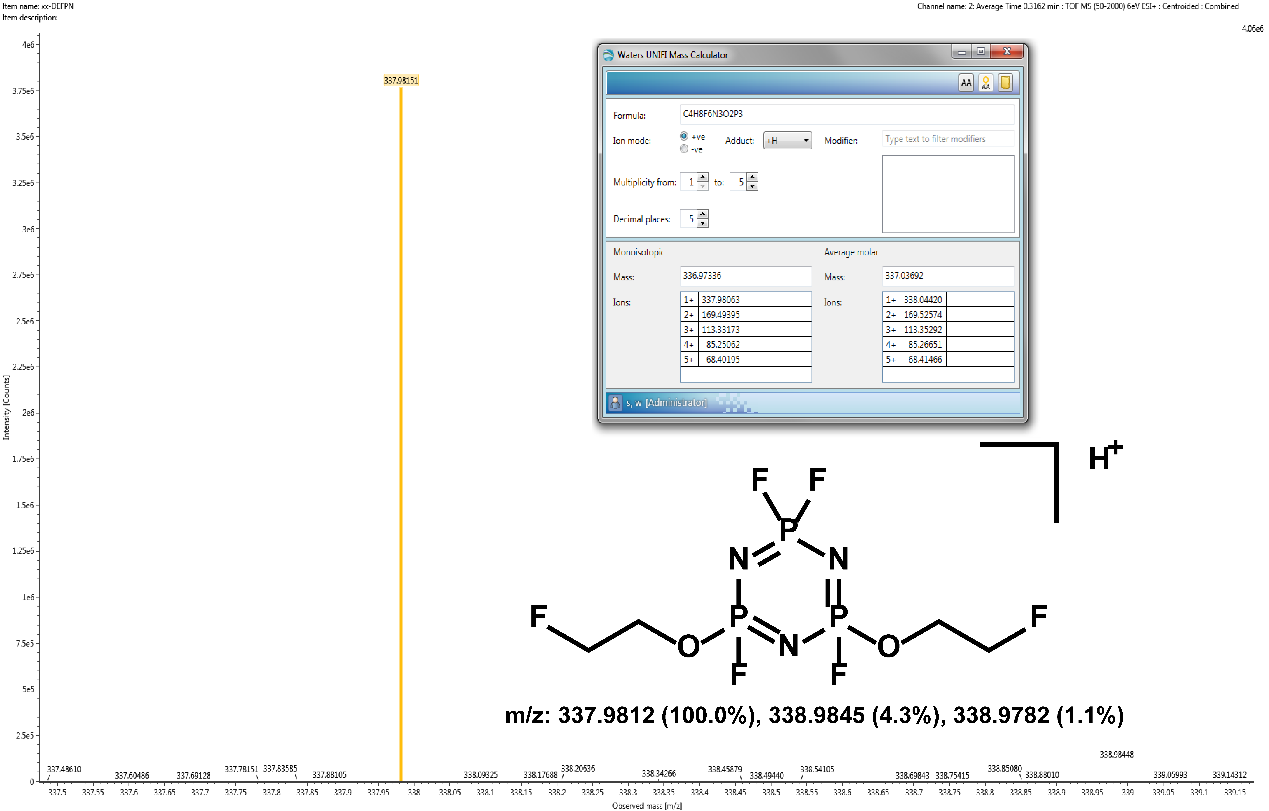


**Figure S13. High resolution mass spectrum of synthesized DFEPN.**

## Physicochemical and electrochemical test

The conductivity was measured by meter (Mettler Toledo LE703) at different temperature.

**Figure S14** illustrates the ionic conductivity of these three electrolytes. The presence of fluorine atoms in PFPN and 2FPN reduces the solubility to lithium salt, resulting in lower ionic conductivity of E-PFPN (10.92 mS·cm^-1^) and E-DFEPN (10.64 mS·cm^-1^) than G2 (11.78 mS·cm^-1^) at 25 ℃. In addition, compared with PFPN, the high content of fluorine atoms in DFEPN further reduces the ionic conductivity. Generally, low conductivity is detrimental to battery performance. However, the difference of ionic conductivity between E-DFEPN and G2 is less than 10%, which has almost no influence on the electrochemical performance under general conditions.

**Figure S14. The ionic conductivity of G2, E-PFPN, E-DFEPN**

The flammability of electrolytes was demonstrated with a direct ignition experiment. Firstly, cotton wool was made into a ball with a diameter of about 1 cm. Then, the cotton ball was placed in electrolyte and soaked for 1 h to fully absorb the electrolyte. Remove the cotton ball completely soaked by the electrolyte, and use the filter paper to absorb the excess electrolyte. Finally, observe the combustion of cotton ball after it was ignited continuously for 5 s with an igniter.

Long-term tests of Li/Cu half cells and Li/Li symmetrical cells were held at a fixed areal capacity of 1.0 mAh·cm^-2^ with a current density of 1.0 mA·cm^-2^, respectively. For Li/LFP half cells and full cells, the cycling performance was performed in a voltage range from 2.5 to 4.2 V (vs. Li/Li^+^) at 0.5 C after three formation cycles at 0.03 C (1 C = 1.62 mA·cm^−2^). The Li/Cu, Li/Li and Li/LFP cells were carried on the electrochemical system (LAND CT2001A, Wuhan, China) at ~298.15 K. Note that the thickness of lithium anode is generally 450 μm, only the thickness is 50 μm in a few cases.

## Electrode characterization

SEM (GeminiSEM 500, China) and transmission electron microscopy (TEM, Talos F200x, America) were employed to characterize the morphology of SEI on Li anode surface and the thickness of CEI on LFP cathode surface, respectively (Figure 4). The elements in SEI and CEI were identified by XPS (Thermo Fisher ESCALAB Xi+, America) with a monochromatized Al Kα X-Ray source. 1H, 31P, 19F-NMR are recorded on JEOL/JNM-ECZ400s/L1，13C-NMR are recorded on AVANCE III HD 600MHz, MS are recorded on Q Exactive Plus.

The composition of SEI on Li anode surface with depth was measured by the argon ion etching. Before experiment, all of the electrode surfaces were washed with DMC three times and dried in the glovebox.

## Contact angle

The optical contact angle measurements were conducted using a DSA100 instrument to evaluate the wettability of different electrolytes on diaphragm (Celgard 3501). The measurement process involved applying homovolumetric droplet of each liquid (50 μL) onto the surface of the diaphragm and capturing images to determine the contact angles.

The contact angle of E-DFEPN (58.3°) is slightly larger than that of G2 (54.1°) and E-PFPN (55.2°), but the difference between the three electrolytes can be negligible (**Figure S15**). Although the addition of flame retardants DFEPN to G2 slightly reduces the wettability of the electrolyte to the diaphragm. Other electrochemical properties of DFEPN make up for the compatibility with interphase.

**Figure S15. The optical contact angle of G2, E-PFPN, E-DFEPN**

## Computational details

The LUMO and HOMO energy of molecule were calculated by adopting density functional theory (DFT) with the Lee-Yang-Parr correlation functional (B3LYP) at 6-311+G (d, p) level.

# Extended discussion

## Random sampled results

**Figure S16** shows partial sampled results by ARECVGA. Because we perform the sampling around zero-point of the prior distribution space, the results exhibit structural similarities: the molecules always have the ether and nitrile sub-structure.


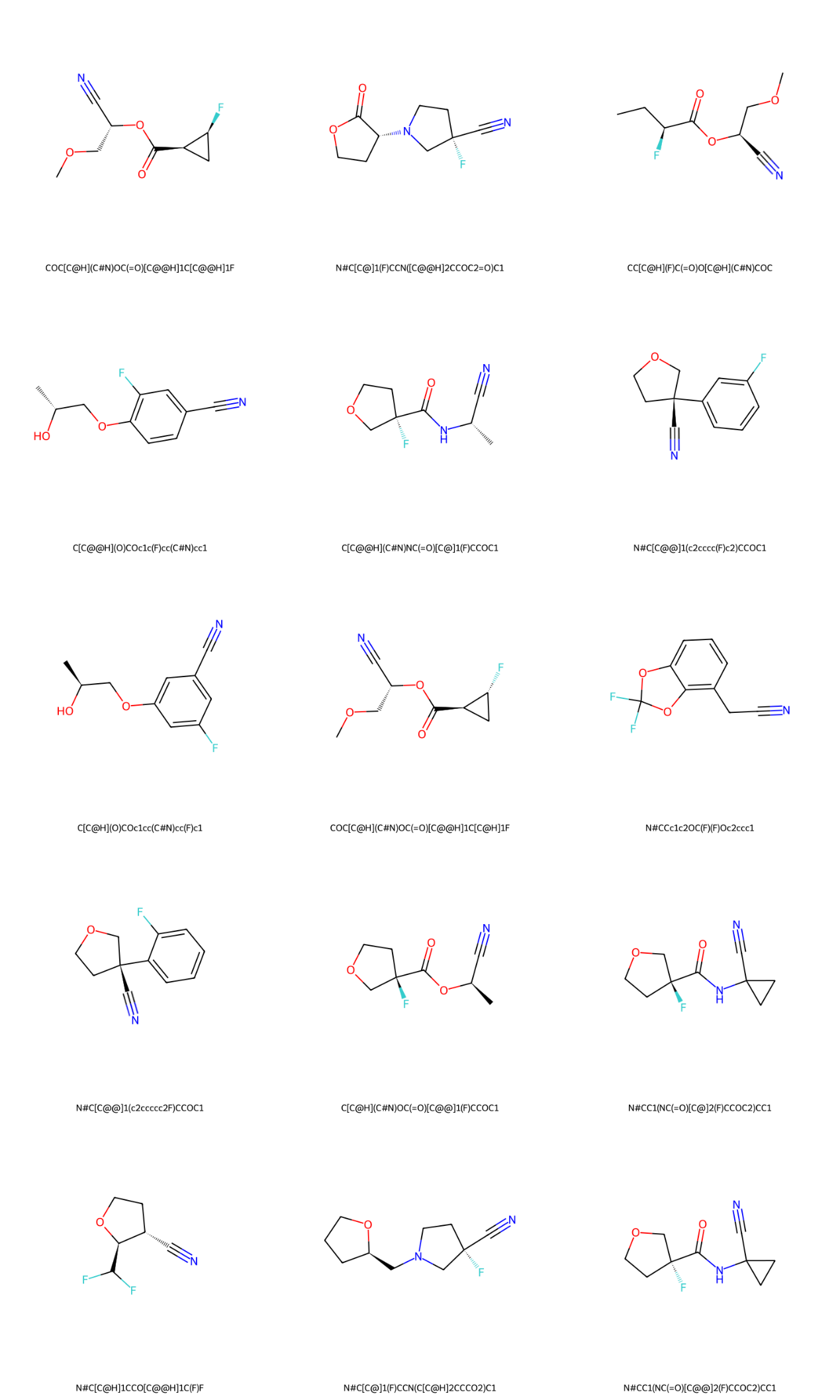


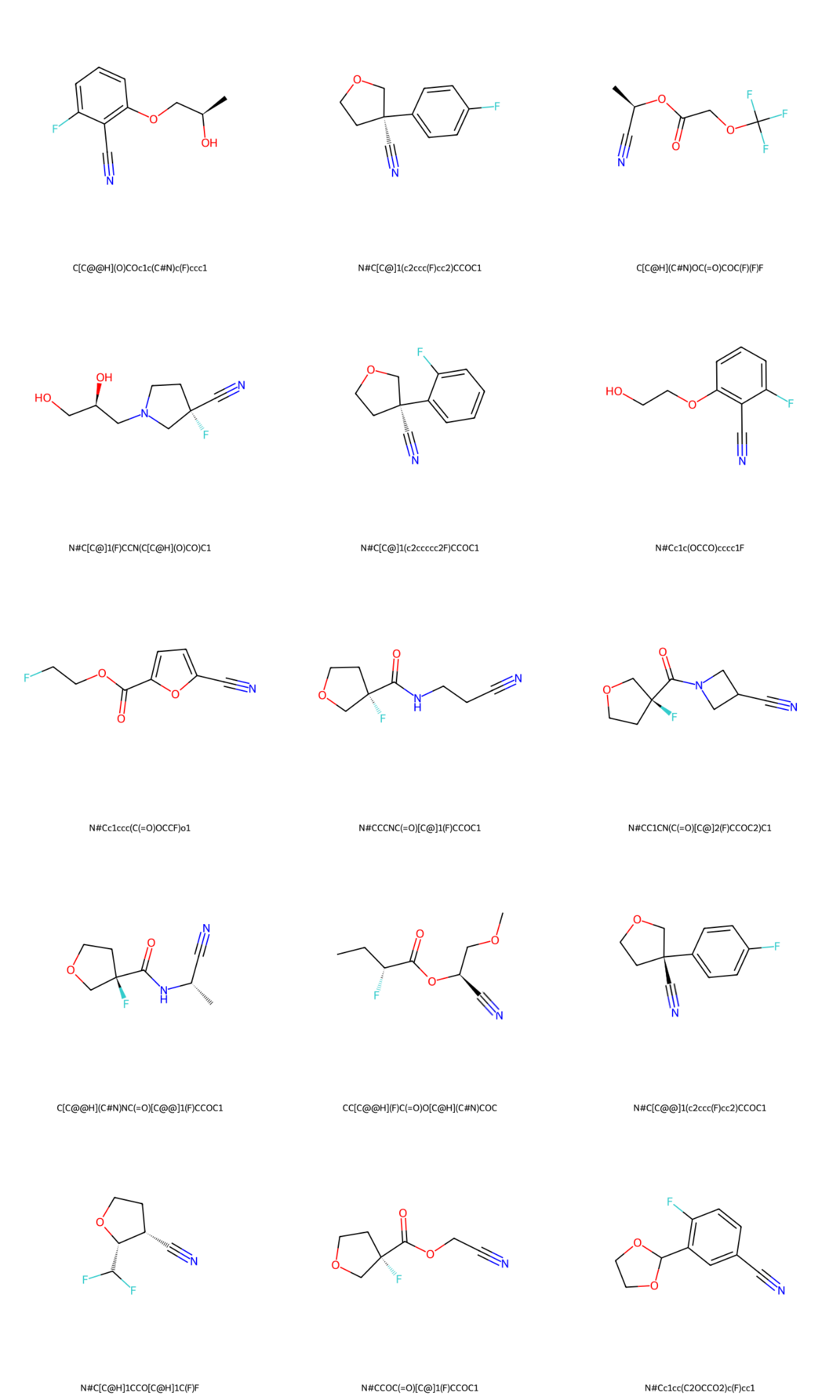

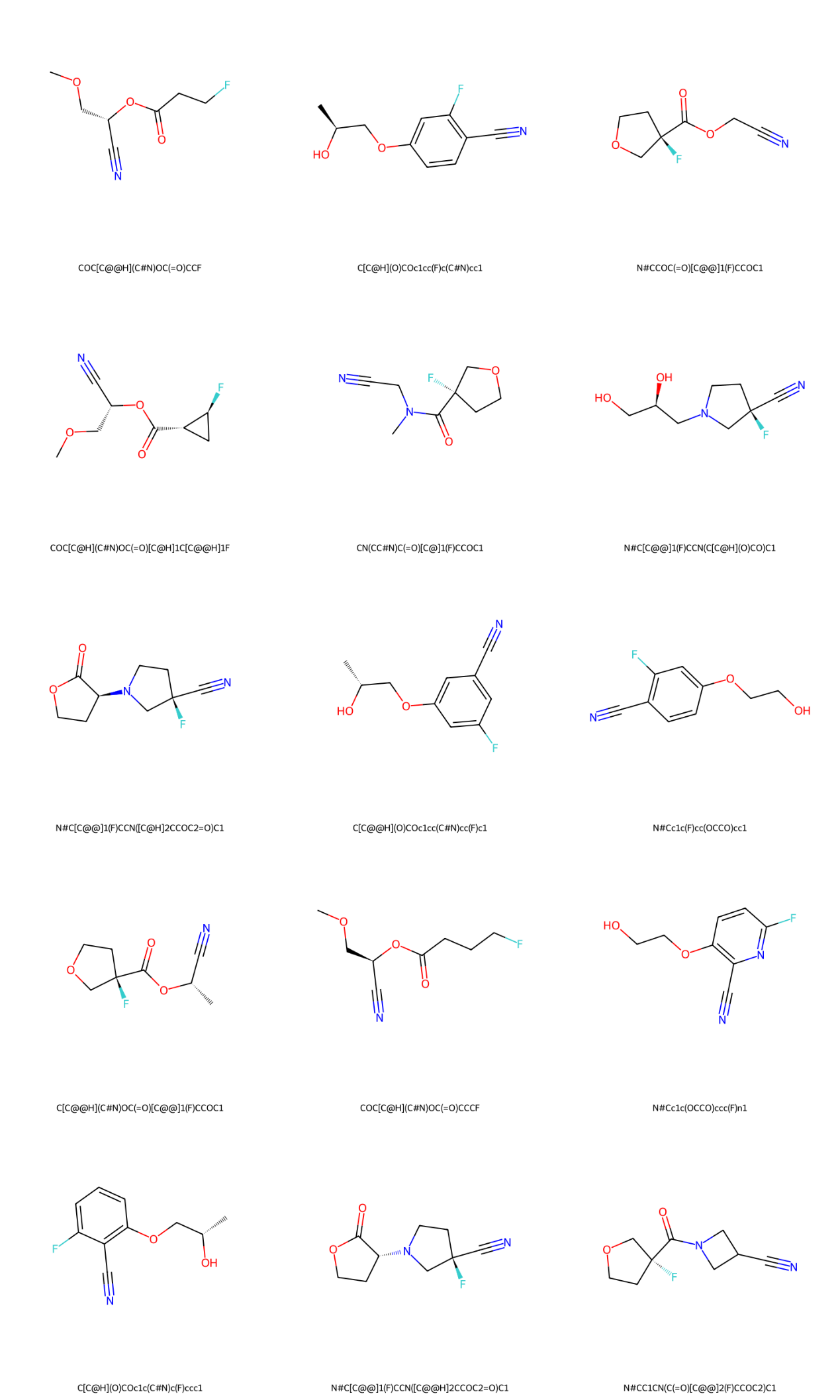

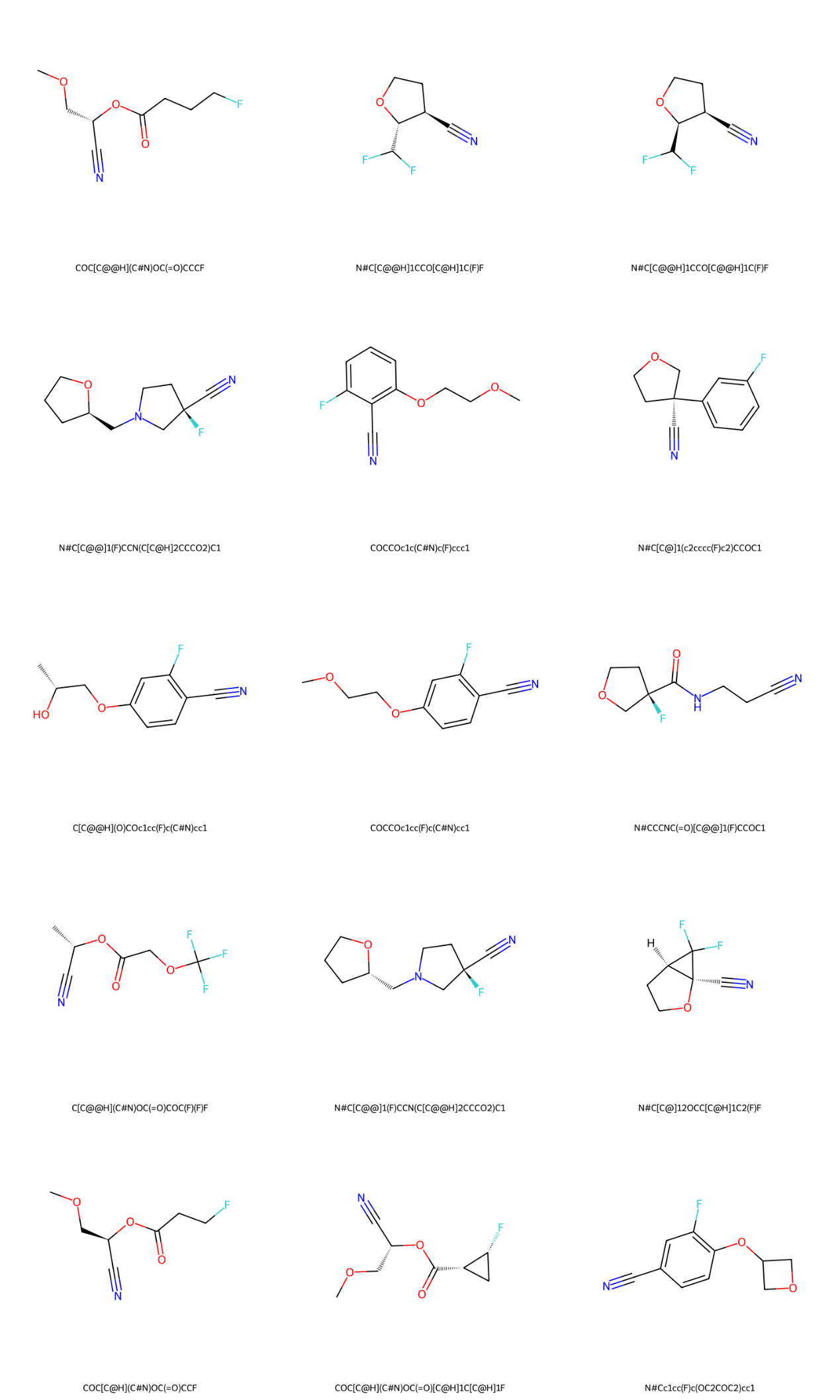

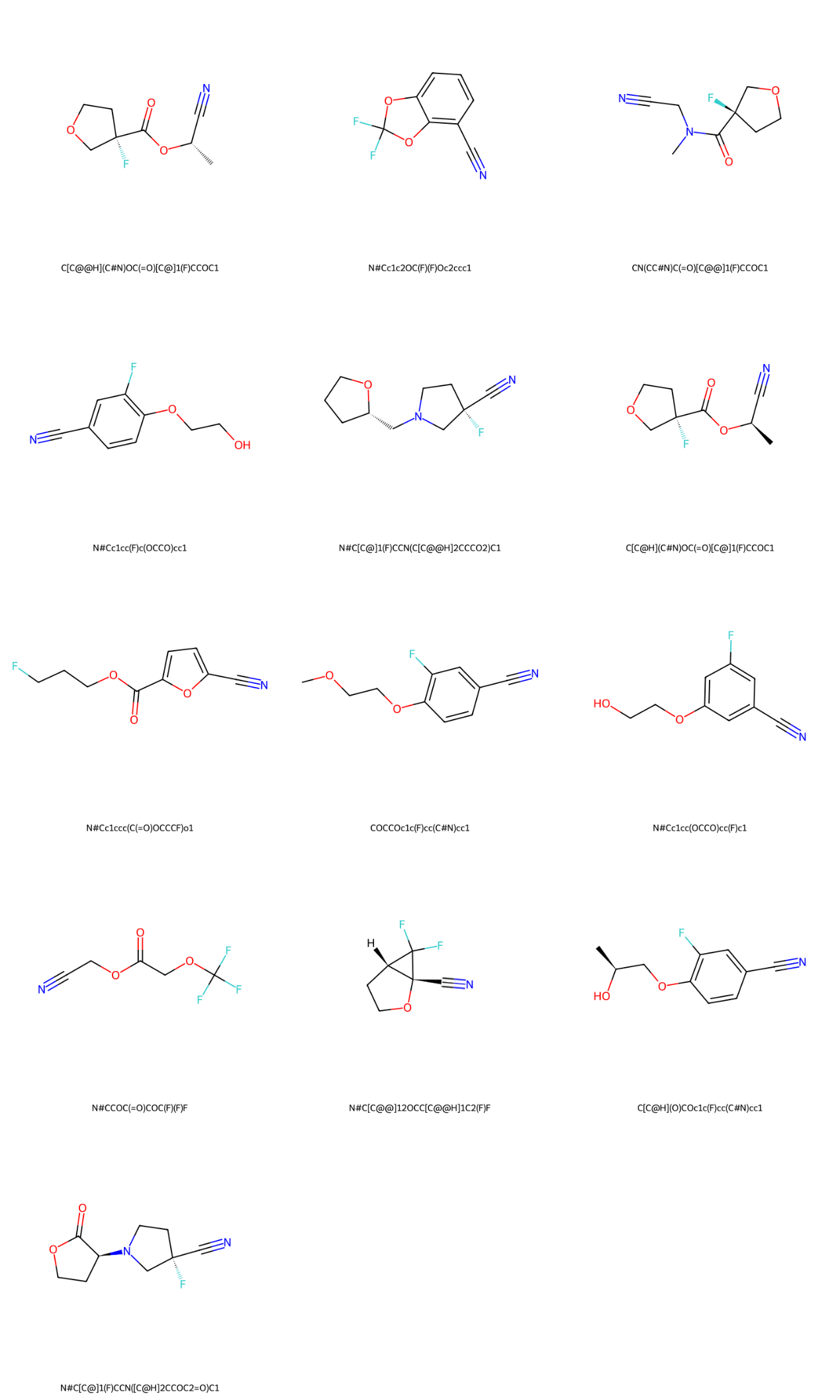


**Figure S16. Random sampled molecules by ARECVGA**

## Generated results of the model

**Figure S17** shows some generated results, the reason of choosing DFEPN for further analysis is mainly because it is gained with highest performance score, and its similar structure with PFPN which makes it possible to be synthesized.


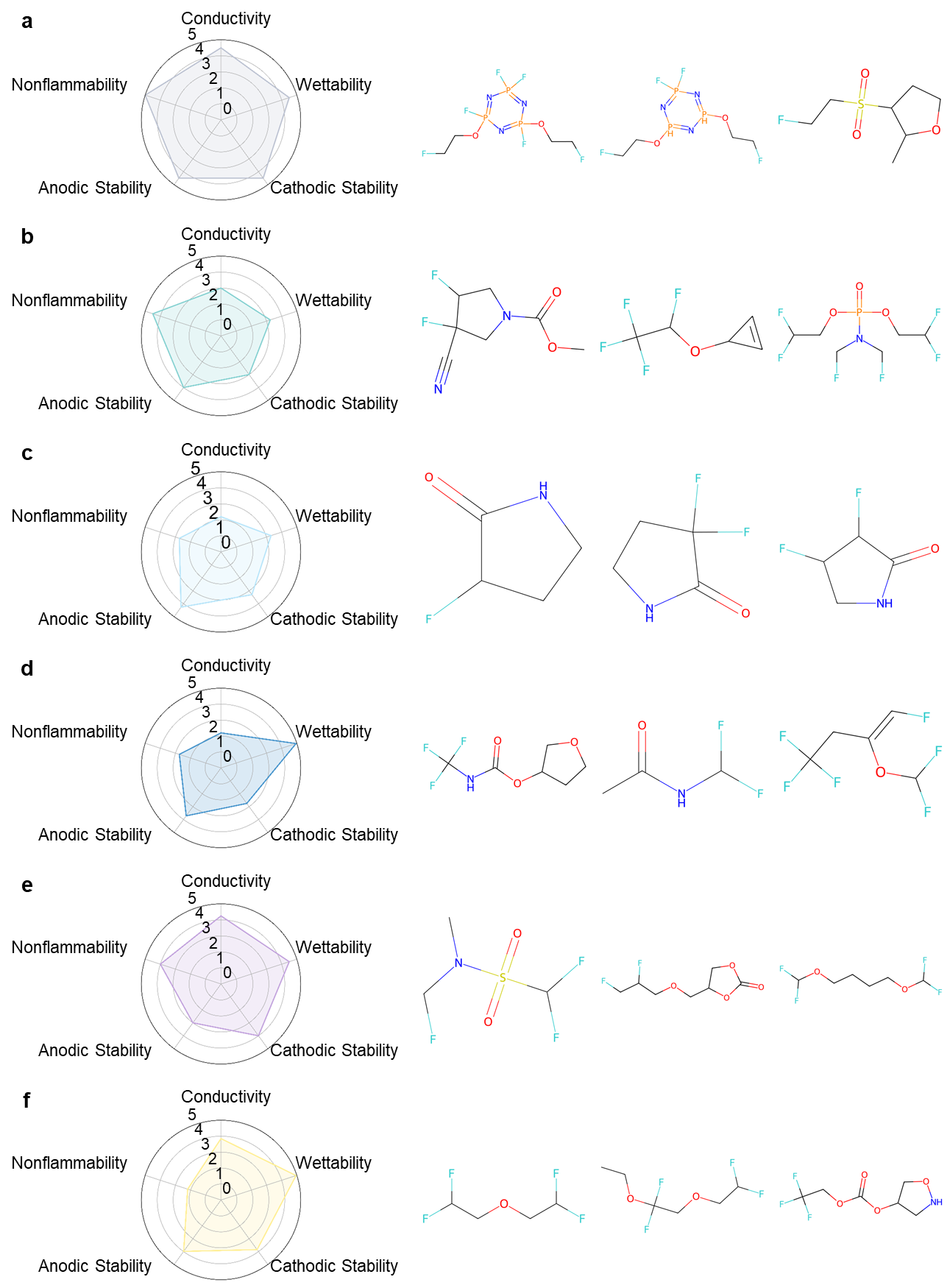


**Figure S17. Some generated results in different generating conditions**

## Model Performance

In this section, we evaluate the model from three key aspects: validity, uniqueness, and novelty, which represent the generative efficiency of the generative models. Validity refers to the ratio of valid molecules to the total number of generated results. Uniqueness is defined as the ratio of unique valid molecules to the total number of generated valid molecules. Novelty measures the proportion of new molecules, those not present in the training data, to the total number of generated valid molecules. To conduct this evaluation, we randomly sampled 10^4^ molecules and compared the results with those from other generative models trained using the ZINC database.

**Table S3. The GuacaMol metrics comparation between models**

| Metric | GraphVAE^[2]^ | MolGAN^[3]^ | NAGVAE^[4]^ | **ARECVGA** |
| --- | --- | --- | --- | --- |
| Validity | 0.140 | 0.017 | 0.926 | **1.000** |
| Uniqueness | 0.316 | 0.201 | 0.614 | **0.688** |
| Novelty | 1.000 | 1.000 | 1.000 | 1.000 |

From **Table S3**, we can conclude that our model efficiently samples novel molecules. The incorporation of atom coordinates as newly selected features and the use of a trainable prior distribution significantly enhance the model's performance. These improvements facilitate subsequent optimizations and conditional sampling processes.

In this work, several efforts, such as the adversarially regularized distribution, ALD, and AEGNN are used for improving model performance, **Table S4** shows how these efforts influence to the model performance. Where , , AR. represent normal distribution, uniform distribution, and adversarially regularized distribution, async. and sync. represent asynchronous and synchronous decoding, respectively. The performances of model in this work (AR., ALD, AEGNN) are bolded. The adversarially regularized distribution performs higher novelty and the ALD performs higher validity, at the same time, the AEGNN-based models usually have better performance than graph convolution networks (GCN)-based models due to its information extraction ability.

**Table S4. The GuacaMol metrics comparation between ARECVGAs with different structures**

| Validity | | | | |
| --- | --- | --- | --- | --- |
| Basic structure | Decode method | Distribution | | |
|  |  | AR. |  |  |
| AEGNN | ALD | **1.000** | 0.895 | 0.874 |
|  | Async. | 0.887 | 0.742 | 0.721 |
|  | Sync. | 0.341 | 0.258 | 0.329 |
| GCN | ALD | 0.855 | 0.753 | 0.714 |
|  | Async. | 0.687 | 0.457 | 0.478 |
|  | Sync. | 0.299 | 0.271 | 0.243 |
| Uniqueness | | | | |
| Basic structure | Decode method | Distribution | | |
|  |  | AR. |  |  |
| AEGNN | ALD | **0.688** | 0.642 | 0.647 |
|  | Async. | 0.654 | 0.452 | 0.574 |
|  | Sync. | 0.423 | 0.454 | 0.346 |
| GCN | ALD | 0.612 | 0.577 | 0.652 |
|  | Async. | 0.668 | 0.512 | 0.627 |
|  | Sync. | 0.583 | 0.574 | 0.426 |
| Novelty | | | | |
| Basic structure | Decode method | Distribution | | |
|  |  | AR. |  |  |
| AEGNN | ALD | **1.000** | 0.898 | 0.857 |
|  | Async. | 0.985 | 0.843 | 0.887 |
|  | Sync. | 0.937 | 0.843 | 0.828 |
| GCN | ALD | 0.952 | 0.812 | 0.875 |
|  | Async. | 0.959 | 0.835 | 0.876 |
|  | Sync. | 0.937 | 0.958 | 0.952 |

## Gradient experiments

The flammability tests (**Figure S18**) demonstrate a critical concentration threshold: electrolytes containing ≥5 wt% DFEPN achieved immediate flame extinction (self-extinguishing time = 0 s) upon ignition source removal, fulfilling non-flammability criteria.

**

**Figure S18. Ignition experiments with electrolytes containing different mass fraction of DFEPN**

Notably, while 10 wt% DFEPN exhibited equivalent flame suppression, its electrochemical performance revealed inherent limitations (**Figure S19**). The 5 wt% formulation demonstrated optimal balance, maintaining a high average Coulombic efficiency (CE) of 91.8% over 200 Li plating/stripping cycles - surpassing both the 3 wt% system (91.5% at cycle 200) and the 10 wt% counterpart that suffered progressive CE decay (post-120 cycles). This confirms 5 wt% as the optimal concentration where flame retardancy enhancement and interfacial stability are maximally preserved.

**

**Figures S19. Long-term Li deposition/stripping CE of electrolytes containing different mass fraction of DFEPN in Li/Cu half cells at 1.0 mA·cm^-2^ and 1.0 mAh·cm^-2^**

## Accelerating Rate Calorimetry (ARC) tests

The baseline electrolyte G2 exhibited an onset temperature of 90.08 °C (**Figure 3**), corresponding to interfacial collapse-induced exothermic reactions. Notably, E-DFEPN demonstrated remarkable thermal stability enhancement, delaying the onset temperature to 170.08 °C. This critical delay provides essential early-warning time for thermal management systems. Additionally, the maximum self-heating rates of E-DFEPN decreases, indicating reduced exothermic reaction intensity. Such moderated heat generation fundamentally lowers thermal propagation risks in practical battery configurations. Eventually, all systems reached ~500 °C (cell venting temperature dictated by mechanical integrity).

The ARC results comprehensively validate that DFEPN modifications not only enhance flame retardancy but crucially improve intrinsic thermal safety through multiple protection mechanisms: delayed onset of exothermic reactions and reduced heat release rates.

Our multi-dimensional analysis (flammability + ARC) establishes a robust safety evaluation framework, confirming DFEPN's superiority in both preventing ignition and suppressing thermal escalation.

**Figure S20. The ARC result** of G2 (**a**) and E-DFFPN (**b**).

## Temperature-dependency of nonflammability

In order to analyze the temperature-dependency of the nonflammability of DFEPN, the heat release rate (HRR) and total heat release (THR) of E-DFEPN at different heat fluxes (5 and 10 kW·m^-2^) were measured by cone calorimetry^[5]^. As shown in **Figure S21,** when the heat flux is increased from 5 to 10 kW·m^-2^, the peak heat release rate (pHRR) of E-DFEPN increases from 160.9 to 186.5 kW·m^-2^ with an increase of 15.9%; The THR increases from 11.4 to 17.3 MJ·m^-2^ with an increase of 51.8%. Notably, under both 5 kW·m^-2^ and 10 kW·m^-2^ radiative heat flux exposures, the material demonstrates stable ignition characteristics with nearly identical onset combustion times of approximately 29 seconds. This observation indicates that the thermal triggering threshold of E-DFEPN remains unaffected by the tested heat flux intensification within this parameter range, highlighting its robust flame-retardant stability against varying thermal intensities.

**Figure S21. a,** HRR **and b,** THR **of E-DFEPN at different heat fluxes of 5 and 10 kW·m^-2^**

## Temperature-dependency of electrochemical performance

As the temperature increases from 25 ℃ to 50 ℃, the capacity retention of Li|E-DFEPN|LFP full cell only decreases from 83.2% to 74.9% after 50 cycles, while the average CE is still as high as 91.4%. It shows that E-DFEPN features exceptional high-temperature cycling performance, which is attributed to the improved thermal stability of E-DFEPN. While, the Li|G2|LFP full cell achieves a low discharge capacity of 30.8 mAh·g^−1^ (20.4% capacity retention) with an average CE of 54.9% after 30 cycles at 0.5 C, showing a poor high-temperature cycling performance.

**Figure S22. The electrochemical performance of Li/LFP full cell at 50 ℃.**

## Extended figures

**
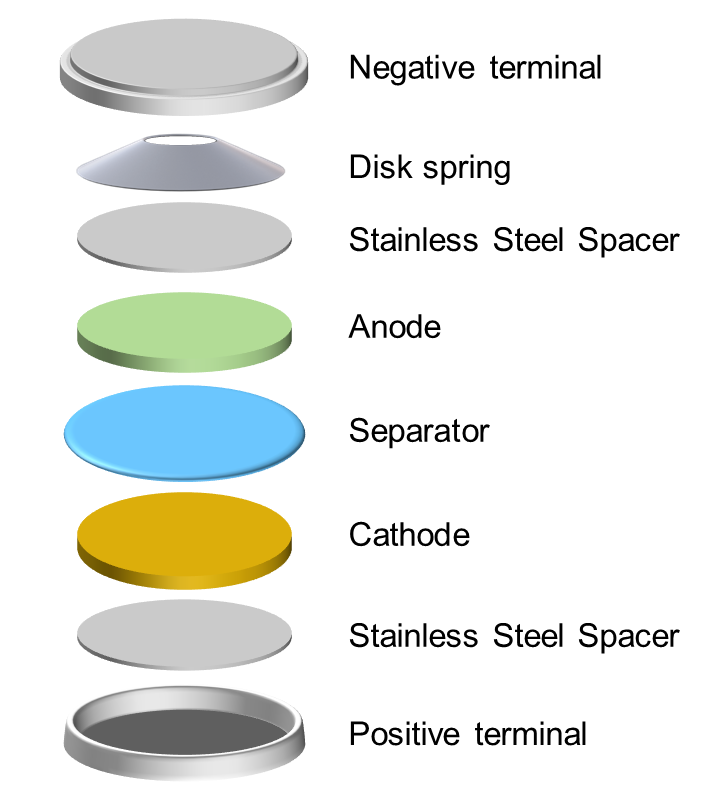
**

**Figure S23. Diagram of coin battery architecture**


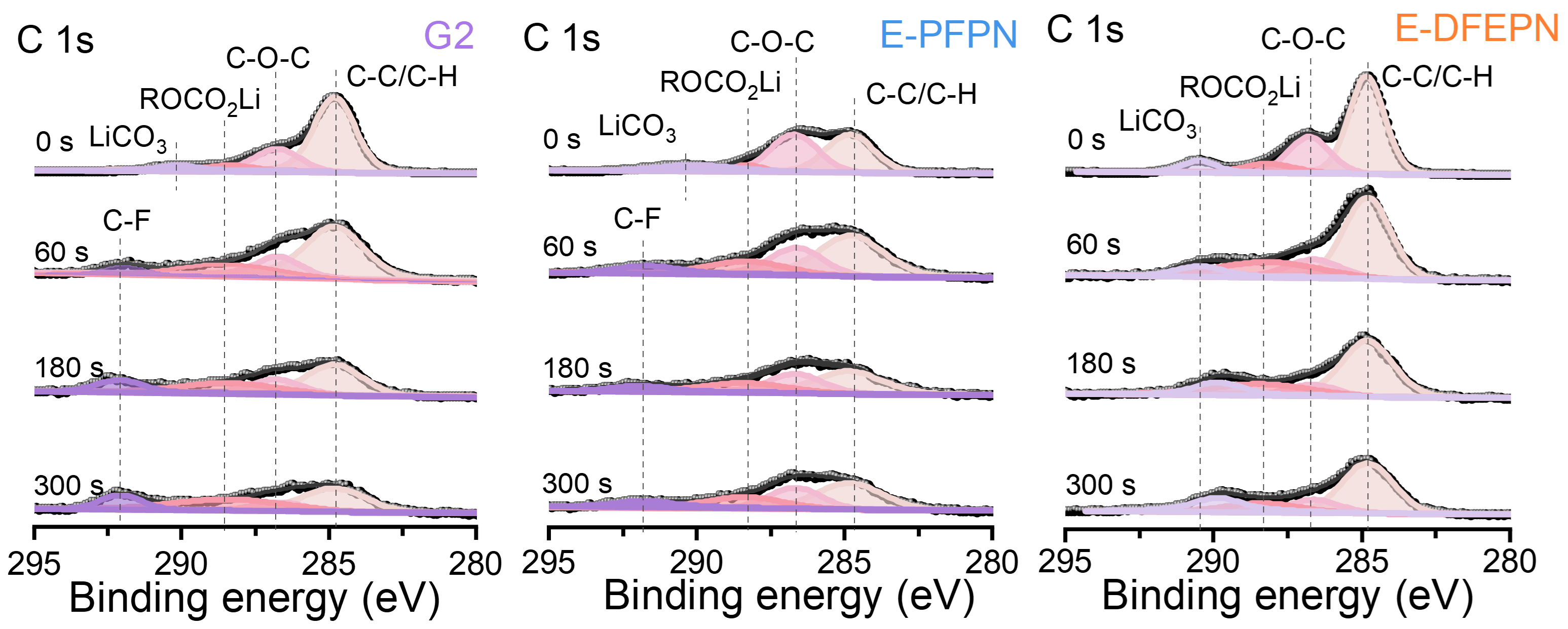


**Figure S24. C 1s XPS spectra of SEI** **formed in Li anode surface after 100 cycles with G2, E-PFPN and E-DFEPN electrolytes before and after 60 s, 180 s, and 300 s etching.**

**Figure S25. The coulombic efficiency of Li/LFP half cells with different electrolyte.**


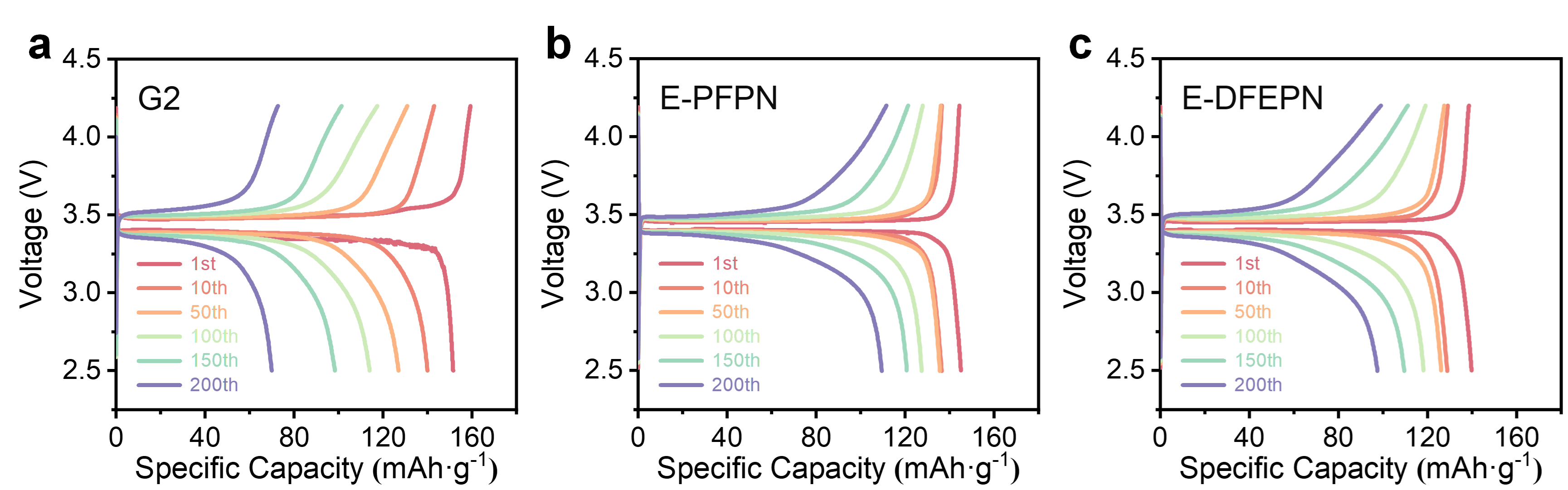


**Figure S26. The charge and discharge profiles in Li/LFP half cells with** **a,** G2, **b,** E-PFPN and **c,** E-2FPN electrolytes.

**Figure S27.The coulombic efficiency of Li/LFP full cells with different electrolyte.**


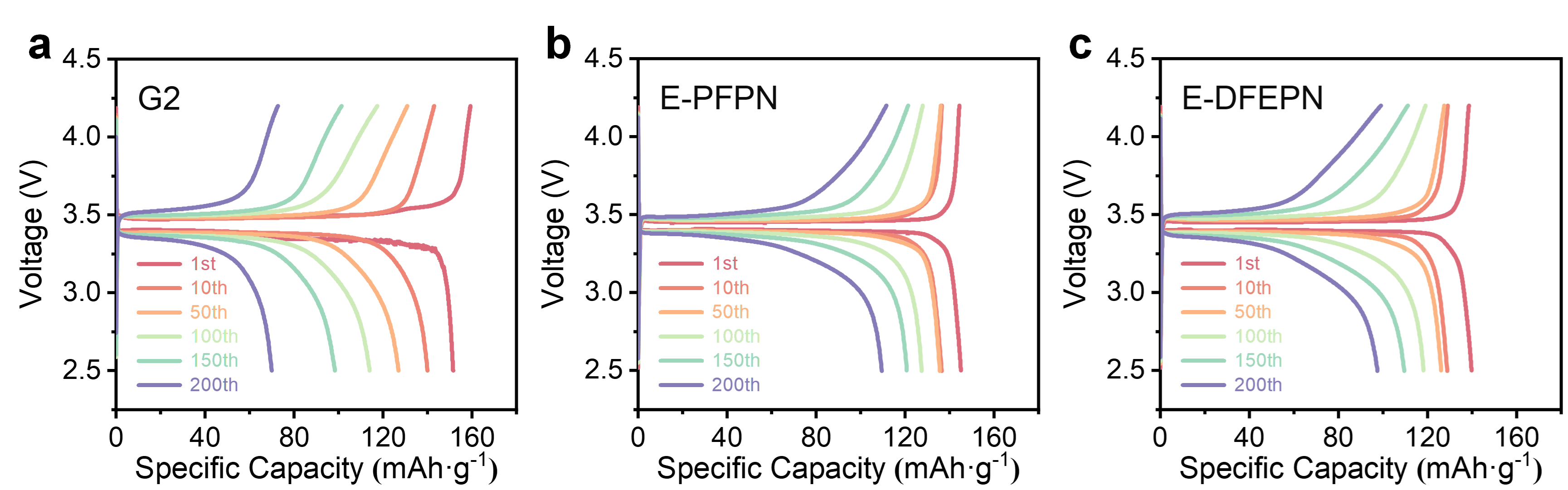


**Figure S28. The charge and discharge profiles in Li/LFP full cells with** **a,** G2, **b,** E-PFPN and **c,** E-2FPN electrolytes.

**Figure S29.** **Long-term cycling performance of** Li/LCO half (**a**) and Li/LCO full (**b**) cells **in a voltage range from 3.00 to 4.45 V.**

# References

[1] L. Greg, T. Paolo, K. Brian, rdkit. Release_2023_09_3 ed.; **2023**.

[2] M. Simonovsky, N. Komodakis, in *Artificial Neural Networks and Machine Learning – ICANN 2018* (Eds.: V. Kůrková, Y. Manolopoulos, B. Hammer, L. Iliadis, I. Maglogiannis), Springer International Publishing, **2018**, 412-422.

[3] N. De Cao, T. Kipf, presented at *International Conference on Machine Learning 2018*, Stockholm, SWEDEN May 01, 2018 **2018**.

[4] Y. Kwon, J. Yoo, Y. S. Choi, W. J. Son, D. Lee, S. Kang, *Journal of Cheminformatics* **2019**, *11* (1), 70, https://doi.org/10.1186/s13321-019-0396-x.

[5] a) A. Yusuf, V. Sai Avvaru, J. De la Vega, M. Zhang, J. Garcia Molleja, D.-Y. Wang, *Chemical Engineering Journal* **2023**, *455*, 140678, https://doi.org/https://doi.org/10.1016/j.cej.2022.140678; b) A. Yusuf, D.-Y. Wang, *Advanced Materials Technologies* **2022**, *7* (6), 2101055, https://doi.org/https://doi.org/10.1002/admt.202101055.
